# Stimuli-Responsive Phenanthrene–Naphthol Fluorescent Marker for Security and Forensic Authentication

**DOI:** 10.1021/acsomega.6c06304

**Published:** 2026-10-21

**Authors:** Rakshitha K Jain, Anand P J, Dhanya Sunil, Poornima Bhagavath, Anoop Kishore Vatti, Shamanth Yagati Udaykumar

**Affiliations:** † Manipal Institute of Technology, 76793 Manipal Academy of Higher Education, Manipal, Karnataka 576104, India; ‡ Future Products Industrial Company, Dammam 31411, Kingdom of Saudi Arabia

## Abstract

Smart functional materials with tunable optical features are highly attractive for anticounterfeiting and forensic applications. In this study, a phenanthrene–naphthol hybrid (PNH) was successfully synthesized and structurally characterized. PNH exhibited good solid-state fluorescence, photostability, intramolecular charge transfer characteristics, solvent-responsive luminescence, and aggregation-induced emission, along with reversible acido- and mechanofluorochromism. Quantitative pressure-dependent studies revealed a progressive and reproducible fluorescence response of PNH to mechanical stimuli. An eco-friendly water-based security ink with PNH as functional pigment was formulated, and the screen-printed patterns exhibited excellent UV stability and abrasion resistance. The prints enabled multimodal authentication through UV-induced fluorescence, reversible mechanical and acid/base vapor switching, and solvent-induced rear-side fluorescence. Notably, the chromic responses retained >90% of their initial fluorescence after 50 switching cycles, demonstrating excellent reversibility and operational durability. Further, PNH enabled high-contrast visualization of latent fingerprints on nonporous, semiporous, complex, and same-background substrates, facilitating the identification of level 1–3 ridge characteristics. Grayscale intensity profiling and three-dimensional surface mapping further confirmed enhanced ridge–furrow contrast and distinct ridge–valley morphology. Molecular dynamics simulations and spectroscopic analysis revealed favorable supramolecular hydrogen bonding and other noncovalent interactions between PNH and representative fingerprint residues, supporting its effective adhesion to latent-print ridges. Its forensic utility was further demonstrated for aged prints and samples exposed to elevated temperature and high relative humidity, where characteristic ridge and groove features remained discernible despite reduced fluorescence intensity. In addition, PNH facilitated clear visualization of groove patterns and minutiae in latent lip prints, indicating significant promise for cheiloscopic analysis. Comparative assessment with recently reported multifunctional fluorescent materials highlights the unique integration of AIE, reversible mechano- and acidofluorochromism, photostability, security-printing capability, and latent finger- and lip-print visualization in a single PNH fluorophore. Overall, PNH represents a versatile platform for next-generation security printing, anticounterfeiting, and forensic latent-print visualization.

## Graphical abstract

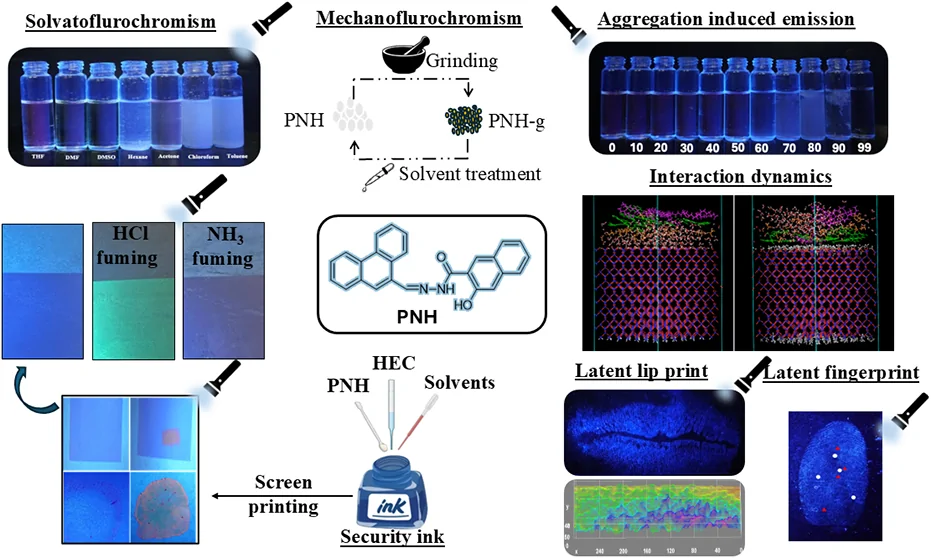

## 1 Introduction

The advancement of high-performance luminescent materials has become increasingly vital in addressing emerging challenges in security printing and forensic science, particularly in anticounterfeiting, as well as latent fingerprint (LFP) and latent lip-print (LLP) detection.^1,2^ Security printing helps prevent counterfeiting, protecting brands, consumers, profits, and regulatory compliance, enabling product verification, combating IP theft, and blocking counterfeit items in the supply chains. Conventional security features such as barcodes, holograms, and watermarks often exhibit limited complexity and are therefore easily replicated and counterfeited.^3,4^ However, luminophores provide multilevel protection for banknotes, documents, and product labels against counterfeiting through their fluorescence, phosphorescence, upconversion emission, and stimuli-induced absorption/emission changes, facilitating multimodal authentication. Similarly, conventional fingerprint and lip-print development methods suffer from low sensitivity, poor ridge contrast, background interference, limited selectivity, toxicity concerns, and potential damage to ridges.^5,6^ All of the above problems are compounded by the fact that finger and lip prints are affected by partial impressions, nonlinear distortions, and small deposition areas.^7,8^ Hence, there is a continual need for smart-performance fluorophores with greater sensitivity and durability to help detect and visualize latent prints on a variety of surfaces found at crime scenes.^1,9,10^

In this context, fluorophores that display aggregation-induced emission (AIE) have gained significant interest owing to their strong solid-state emission, making them suitable as functional pigments in security inks and forensic visualization.^5,11^ Phenanthrene–naphthalene hydrazone derivatives represent a promising class of AIE-active luminogens due to their extended conjugation and intramolecular charge transfer (ICT) behavior.^12,13^ Their molecular architecture enables excellent photostability and emission tunability, supporting multicolor security printing and encrypted luminescent features^12,14,15^ that are difficult to replicate.^16,17^ Hydrazone- and Schiff-base-based AIE frameworks have been widely explored as fluorescent fingerprinting powders and fluorescent security markers, highlighting their potential in forensic and anticounterfeiting applications.^2,18^ Phenanthrene-derived luminogens have demonstrated multimodal anticounterfeiting performance and TNT sensing, while naphthalimide-, imidazole-, and naphthalene-based AIEgens show selective detection of phenolic nitro-explosives in aqueous media,^19,20^ scalable low-cost anticounterfeiting solutions,^5,21^ and offer light-up responses toward volatile chlorinated solvents such as chloroform.^22^ Alongside AIE and photostable fluorophores, stimuli-responsive luminescence is increasingly recognized as a crucial feature for next-generation security technologies. In particular, mechanofluorochromism, characterized by reversible fluorescence switching under mechanical stimulation, such as grinding, pressing, or shearing, offers an attractive platform for security and forensic applications.^23,24^ This response arises from changes in molecular packing, crystalline–amorphous transitions, or conformational rearrangements.^11,25^ Accordingly, mechanoresponsive hydrazone- and Schiff-base-derived fluorophores including tetraphenylethene hydrazones, naphthaldehyde Schiff bases, phenanthrene derivatives, pyrene-derived amidophosphonates, and phenylene ethynylenes have shown strong potential in pressure sensing, rewritable data storage, and advanced anticounterfeiting, enabling their use as tamper-indicating security materials.^24,26–31^ Such mechanically activatable covert features provide an additional protection layer against forgery.^23^

Despite the rapid development of fluorescent materials for anticounterfeiting and forensic applications, most reported systems are designed for a limited number of functions and often rely on a single stimulus or authentication mechanism. In particular, the integration of AIE, reversible mechano- and acidofluorochromism, photostability, multimodal security printing, and latent finger- and lip-print visualization within a single metal-free fluorophore remains relatively unexplored.^28,32,33^ Moreover, the molecular interactions governing fluorophore adhesion to fingerprint residues and the performance of such materials under challenging forensic conditions have received a limited amount of attention. A systematic comparison of these multifunctional attributes with state-of-the-art fluorescent systems is also lacking, highlighting the need for integrated platforms that combine optical versatility, durability, security functionality, and forensic applicability. In this work, we report a phenanthrene–naphthol hydrazone fluorophore (PNH) as a multifunctional platform for security printing and forensic authentication. PNH integrates AIE, ICT, and reversible mechano- and acidofluorochromism within a single molecular framework and is formulated into an eco-friendly, water-based screen-printing ink for multimodal authentication. Its interactions with fingerprint-residue components are investigated using molecular dynamics simulations and FTIR analysis, while its forensic utility was evaluated through latent finger and lip-print visualization on diverse substrates. Finally, PNH is benchmarked against representative fluorescent systems to assess its multifunctional optical, security printing, and forensic performance, highlighting its potential for next-generation anticounterfeiting and forensic applications.

## 2 Experimental Details

### 2.1 Synthesis of 3-Hydroxy-*N*’-(phenanthren-9-ylmethylene)-2-naphthohydrazide (PNH)

The chemicals used for the synthesis are 9-acetylphenanthrene (Catalogue No. A19601) and 3-hydroxy-2-naphthohydrazide (Product No. H0282), obtained from Sigma-Aldrich and TCI, respectively. Methanol used as a solvent was purchased from Spectrochem Chemicals Pvt. Ltd., and silica gel 60 F254 TLC plates (MS-grade, Merck) were used to track the progress of the synthesis reaction of PNH. The reaction mixture containing 9-acetylphenanthrene (0.242 mmol) and 3-hydroxy-2-naphthohydrazide (0.266 mmol) dissolved in methanol was refluxed at 80 °C in a 100 mL round-bottom flask for 8 h under continuous stirring and cooled to room temperature. The precipitate was collected by filtration, washed with cold methanol, and dried under vacuum. The crude product obtained was recrystallized from methanol to afford PNH (Scheme 1).

**Scheme 1:**
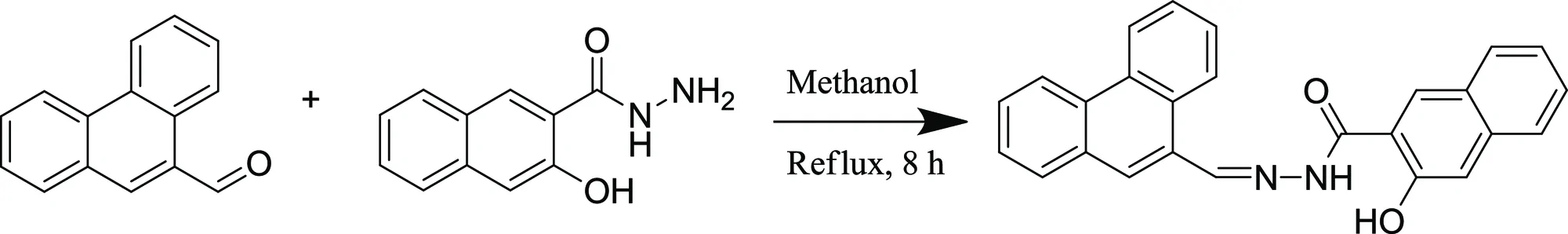
Synthesis Route for PNH

### 2.2 Characterization of PNH

The ATR-FTIR spectrum was obtained using a Shimadzu IR Spirit analyzer (40 scans, 4 cm^–1^ resolution). The NMR spectra were recorded in DMSO-*d*_6_ on a 400 MHz Bruker spectrometer. LC-MS analysis was performed using a Waters liquid chromatography–mass spectrometry system equipped with a photodiode array detector and a single quadrupole mass analyzer. Data acquisition and processing were carried out using MassLynx software (Waters). Chromatographic separation was achieved under isocratic conditions using a mobile phase consisting of 0.05% (v/v) formic acid in water and 0.05% (v/v) formic acid in acetonitrile in a 50:50 (v/v) ratio. The flow rate was maintained at 0.5 mL min^–1^ with an injection volume of 2 μL, methanol as the diluent, and a total run time of 2 min. Mass spectrometric detection was carried out using an electrospray ionization source operated in both positive and negative ion modes (ES^+^/ES^–^). The capillary and cone voltages were set to 1.50 kV and 15 V, respectively. The desolvation temperature was maintained at 600 °C, with a gas flow of 1000 L h^–1^ and a cone gas flow of 50 L h^–1^. About 2 mg of the sample was accurately weighed and transferred into a 10 mL volumetric flask, followed by the addition of 2 mL of methanol. An aliquot of 1 mL of this solution was transferred into an HPLC vial and used for analysis. The melting point was measured by the open capillary method. Photoluminescence (PL) spectra were recorded on a Horiba Fluorolog-QM-75–21 spectrofluorometer, with excitation and emission bandwidths set between 2.5 and 5 nm. Surface morphology studies were performed using a Carl Zeiss EVO 18 analytical scanning electron microscope (SEM), and dynamic light scattering (DLS) analysis for particle size measurement was achieved using a HORIBA Nano Particle Analyzer operated at a scattering angle of 90°. X-ray diffraction (XRD) data was obtained using a Rigaku Ultima IV diffractometer. The diffraction patterns were collected using Cu Kα radiation (λ = 1.540562 Å) with a Ni filter. The samples were scanned over a 2θ range of 5–80° with a step size of 0.02°, at a scanning rate of 2° min^–1^. Acidofluorochromic studies were carried out by exposing PNH to HCl vapor in a sealed chamber at room temperature, followed by PL measurements under identical conditions. The reversibility of the fluorescence response was evaluated by subsequent exposure of the HCl-treated samples to NH_3_ vapor.

### 2.3 Density Functional Theory

The single, dimer, and trimer PNH molecule structures were optimized in vacuum and tetrahydrofuran (THF) solution-phase conditions using DFT in Jaguar,^34,35^ applying the B3LYP^36^ exchange–correlation functional, 6-311G** basis set with dispersion corrections (D3).^37,38^ The solvation effects of THF were described using the polarizable continuum model (PCM)^39,40^ at 298 K, utilizing the Bondi radii.^41^ Following the geometry optimization, the PNH molecules were subsequently employed for electronic properties calculations such as electrostatic surface potential (ESP), ESP atomic charges, and highest occupied molecular orbital (HOMO)–lowest unoccupied molecular orbital (LUMO). The LUMO–HOMO energy gap (Δ*E*) was determined using Δ*E* = *E*_LUMO_ – *E*_HOMO_, where *E*_LUMO_ and *E*_HOMO_ represent the orbital energies of LUMO–HOMO, respectively.

### 2.4 Assessment of PNH Cytotoxicity

The cytotoxicity of PNH was evaluated in HEK293T cells using MTT assay. Approximately 10,000 cells per well were seeded in triplicate into 96-well plates containing 100 μL of DMEM media supplemented with 10% FBS and incubated for 24 h at 37 °C in a 5% CO_2_ atmosphere. Following incubation, the cells were treated with varying concentrations of PNH (DMSO as vehicle control) and maintained for 48 h. After treatment, the wells were washed with fresh medium, and 50 μL of MTT solution (5 mg/mL in PBS) was added. The plates were further incubated for 4 h, MTT solution was removed, and 100 μL of DMSO was added to dissolve the formazan crystals. After gentle mixing and a 20 min incubation in the dark, absorbance at 570 nm was measured using a microplate reader. The IC_50_ value was obtained by plotting the mean percentage reduction in cell viability against increasing concentrations of PNH.

### 2.5 Mechanofluorochromic Studies

To quantitatively evaluate the pressure-dependent mechanofluorochromic response, we compressed PNH into pellets using a pellet press equipped with a 12 mm diameter die. Compressive loads of 2, 4, 6, 8, and 10 N were applied individually, corresponding to nominal pressures of 0.018, 0.035, 0.053, 0.071, and 0.088 MPa, respectively, based on the die area of 113.1 mm^2^. The PL spectra of the pellets were recorded at an excitation wavelength of 390 nm under the same instrumental conditions. The emission spectra were normalized to their respective maximum intensities to facilitate a direct comparison of pressure-dependent spectral changes. The calculated nominal pressures, obtained from the applied load divided by the die area, were used to systematically evaluate the pressure sensitivity of the mechanofluorochromic response.

### 2.6 Formulation of Water-Based Security Ink

A PNH-based screen ink was developed using hydroxyethyl cellulose (HEC: CAS no. 9004-62-0, Sigma-Aldrich) as an eco-friendly binder. Table 1 presents the optimized formulation of the screen ink, wherein the required balance between the binder, solvents, additives, and PNH pigment is maintained to ensure stable dispersion and printability. A 5 wt % aqueous HEC solution was incorporated as a primary component of the formulation, comprising 83 wt % of the ink. This solution served as the binder, providing an appropriate viscosity, maintaining dispersion stability, and ensuring optimal printability. Butyl carbitol (1 wt %) was incorporated in the formulation as a cosolvent, ensuring proper ink drying and minimizing defects in the print. Isopropyl alcohol (5 wt %) was added to the ink formulation, ensuring proper wetting and drying after printing. Preventol (1 wt %) was incorporated as a biocide, preventing the growth of microorganisms in the ink and ensuring storage stability. PNH pigment (10 wt %) was added to the ink, ensuring stable optical quality and print durability.

**Table 1:** Water-Based Eco-Friendly Screen Ink Formulation for Security Printing

component	percentage (wt %)
5% HEC solution	83
butyl carbitol	1
isopropyl alcohol (IPA)	5
preventol	1
PNH	10

### 2.7 Screen Printing of Inks and Study of Print Features

The formulated screen ink was printed on UV-dull paper sourced from Manipal Press Pvt. Ltd., Manipal, India. Screen printing was performed using a 100 T mesh screen, and the ink was printed onto the paper substrate using a squeegee. Screen-printed samples (1.5 cm × 1.5 cm) were exposed to 300 W, 30 m/min UV light from a UV curing unit for 0, 10, 20, 30, 40, and 50 pass cycles to evaluate the optical stability of the ink film. After each exposure cycle, PL spectra were recorded under ambient conditions. The abrasion resistance of the printed ink film was assessed using a Sutherland ink rub tester. Printed and unprinted paper strips (8 cm × 5 cm) were fixed onto the base plate and rubber pad, respectively. A 4 lb weight was placed on the test block, and the samples were subjected to different stroke counts. Print density and colorimetric parameters (L*, a*, b*) were measured using an X-Rite i1 Pro spectrophotometer following the CIE Lab* color system. L* represents lightness (0 = black, 100 = white), while a* and b* values range from −120 to +120, where negative a* and b* correspond to green and blue, and positive values correspond to red and yellow, respectively. The overall color difference (Δ*E*) was used to quantify visual variation, where values of 0–2 are not perceptible to the human eye, 2–3 are detectable by trained observers, and values above 3 are clearly noticeable. The color difference (Δ*E*) was calculated using $\Delta E=\sqrt{{\left(\Delta L*\right)}^{2}+{\left(\Delta a*\right)}^{2}+{\left(\Delta b*\right)}^{2}}$ where (ΔL*) = L*_(*u*)_ – L*, (Δa*) = *a*_(*u*)_^*^ – a*, and (Δ*b*) = *b*_(*u*)_^*^ – b* signify the differences between the unprinted (*u*) and printed paper samples.^42^

### 2.8 Molecular Docking Studies

#### 2.8.1 SiO_2_ Surface Construction

The surface was constructed by considering bulk quartz (α-SiO_2_) as the initial structure. A slab terminating with oxygen, consisting of six-unit cells in thickness, representing the (001) surface, was built. Surface neutralization was achieved by terminating the surface with hydrogen and methyl groups to prepare hydrophilic and hydrophobic surfaces, respectively. The resulting surface unit cell was then expanded into the 7 × 7 supercell along the *x*- and *y*-directions.

#### 2.8.2 MD Simulation Studies

The latest optimized potentials for liquid simulations (OPLS4)^43^ force fields in DESMOND MD code^44^ within the Schrodinger materials science suite were employed in this study.^45^ The 1 fs time step and 9 Å cutoff distance were considered to characterize the nonbonded interactions. A Nosé–Hoover thermostat, with a relaxation time of 1 ps, was employed. The simple point charge (SPC) model was utilized for describing water molecules. Initially, 200 ps at 10 K Brownian minimization followed by 10 ns NVT equilibration run at 300 K was performed and finally, 50 ns NVT ensemble at 300 K production run was performed. Particle Mesh Ewald (PME)^46,47^ was considered for electrostatics. A vacuum region of 25 Å was considered. The number of molecules and respective wt % of the optimized PNH, glycine, myristic acid, and water molecules considered on the surface are summarized in Table 2.

**Table 2:** Molecules Considered on Both Hydrophilic and Hydrophobic Surfaces

sl. no.	molecule	number of molecules	weight (%)
1	glycine	16	12.2
2	myristic acid	6	14
3	water	272	49.9
4	PNH	6	23.9

### 2.9 Cycling Stability of Reversible Mechanofluorochromic and Acidofluorochromic Behavior

The cycling stability and reversibility of chromic behavior of PNH-based prints were evaluated through repeated mechanofluorochromic and acidofluorochromic switching cycles. For the mechanofluorochromic study, the prints were mechanically scratched to induce fluorescence switching, followed by exposure to DCM vapor to restore the initial fluorescence state. In the acidofluorochromic study, the prints were exposed to concentrated HCl vapor to induce fluorescence switching, followed by NH_3_ vapor treatment to recover the initial fluorescence state. A scratch-DCM vapor recovery sequence for the mechanofluorochromic response and an HCl vapor–NH_3_ vapor switching sequence for the acidofluorochromic response is defined as one complete cycle. Each switching process was repeated for 50 consecutive cycles to assess the durability and reversibility of the chromic behavior. PL spectra were recorded after every 10 cycles under identical experimental conditions to monitor any changes in emission intensity and spectral profile during repeated cycling. The fluorescence intensity retention (%) was further calculated ( $\mathrm{Fluorescence intensity retention}\left(\%\right)=\frac{I_{0}}{I_{n}}\times 100$ .

### 2.10 Development and Visualization of LFPs

Latent fingermarks were obtained by gently rubbing fingertips on regions of the skin rich in sebaceous and eccrine secretions, such as the nose and forehead, to closely depict the typical composition of fingermark residues found in forensic investigations. Fingermarks were deposited onto a range of surfaces and subsequently developed using a finely milled PNH powder. Following application, surplus powder was carefully removed with a Marabou forensic brush to enhance the ridge clarity. Processed fingermarks were visualized under 380 nm UV illumination, and images were taken by using an iPhone 14 camera. Further image analyses, such as three-dimensional (3D) surface mapping of fingermarks and gray-level intensity profiling of ridge structures, were performed using ImageJ software. The acquired two-dimensional (2D) grayscale fingermarks were converted into 3D surface maps using the Interactive 3D Surface Plot plugin in ImageJ. In the generated 3D surface, the *x*- and *y*-axes represent the image coordinates (pixels), while the *z*-axis corresponds to the gray value (a.u.), representing the relative fluorescence intensity of each pixel. A color lookup table was applied to enhance the visualization of intensity variations across the fingerprint ridges and furrows. The *z*-axis represents the relative pixel intensity rather than the actual physical ridge height, as the 3D surface was reconstructed from a conventional 2D fluorescence image.

Static water contact angle measurements of representative substrates were carried out using a Theta Flex optical tensiometer (Biolin Scientific, Finland) at room temperature to evaluate the influence of substrate wettability on LFP development. Glass, glossy paper, compact disc (CD), ceramic tile, and titanium were selected as substrates for the study. A 5 μL droplet of deionized water was carefully deposited onto each substrate surface, and the static contact angle was recorded immediately after droplet placement. Measurements were performed at three different locations on each substrate, and the average contact angle values are reported as mean ± standard deviation (SD). The surface roughness of the CD substrate was analyzed using a 3D Non-Contact Profilometer (ST400, NANOVEA). The measured wettability and roughness parameters were correlated with the LFP visualization performance of PNH.

### 2.11 Development and Visualization of LPPs

For lip impressions, the lips were swabbed, and the impressions were deposited on clean glass substrates by pressing the lips for 3–4 s. The impressions were developed using PNH powder, followed by the removal of excess powder with a Marabou feather brush and visualized under 380 nm UV irradiation. Images were captured by using an iPhone 14 camera. Grayscale intensity profiling and 3D surface mapping were performed by using ImageJ in the same manner as described for LFPs. The fluorescence images were further processed by grayscale conversion and contrast enhancement using GIMP to facilitate the identification of lip-print features.

### 2.12 Development of Aged Prints under Different Environmental Conditions

To evaluate the practical applicability of PNH for forensic investigations, LFPs and LLPs were deposited by the same volunteer onto clean glass substrates under identical conditions. The volunteer was instructed not to apply hand lotion, moisturizer, lipstick, lip balm, or any cosmetic products prior to sample collection. Freshly deposited prints were stored in an undeveloped state under ambient laboratory conditions (25 ± 2 °C and 50–60% relative humidity (RH)) for 1 and 20 days to investigate the effect of aging on the fingermark visualization performance of PNH. The latent prints were then developed by gently dusting with PNH powder using a Marabou forensic brush, and excess powder was carefully removed before imaging under UV illumination. In addition, freshly deposited undeveloped latent prints were separately exposed for 24 h under elevated temperature (70 °C) in a laboratory oven and high-humidity conditions (92% RH), maintained using a saturated potassium nitrate (KNO_3_) solution. Following environmental exposure, the latent prints were developed with PNH using the same dusting procedure and subsequently photographed under identical UV illumination and camera setting.

## 3 Results and Discussion

### 3.1 Structural Characterization of PNH

The structure of the acyl hydrazone derivative PNH was confirmed through spectroscopic analysis. FTIR showed characteristic C=O and C=N stretching bands as shown in Figure S1. The ^1^H NMR spectrum exhibited a singlet at 8.96 ppm (imine proton), signals for 15 aromatic protons, and −OH/–NH groups (Figure S2), indicating 97.82% purity. The ^13^C NMR spectrum displayed signals for 26 carbon atoms (Figure S3), while mass spectrometry exhibited a base peak at *m*/*z* = 391 (M+1), further supporting the proposed molecular structure of PNH (Figure S4).

White powder (% yield: 94); mp: > 250 °C; FTIR (cm^–1^): 3450–3600, 1642, 1554, 1454; ^1^H NMR (400 MHz) δ: 12.16 (s, 1H, −OH), 11.34 (s, 1H, –NH), 9.15–9.11 (m, 2H), 8.96 (s, 1H, CH = N), 8.90–8.88 (d, 1H, 8 Hz), 8.53 (s, 1H), 8.32 (s, 1H), 8.17–8.15 (d, 1H, 8 Hz), 7.97–7.95 (d, 1H, 8 Hz), 7.81–7.79 (d, 4H), 7.74–7.70 (t, 1H, 8 Hz), 7.56–7.52 (t, 1H, 8 Hz), 7.41–7.33 (m, 2H) (97.82% purity); ^13^C NMR (100 MHz) δ: 164.31, 154.59, 149.34, 136.37, 131.08, 131.04, 130.83, 130.77, 130.53, 129.88, 129.28, 129.17, 128.84, 128.75, 128.68, 128.01, 127.86, 127.75, 127.30, 126.36, 125.93, 124.33, 124.02, 123.45, 120.92, 111.10; MS (ESI): *m*/*z* for C_26_H_18_N_2_O_2_ 390.43 (calc.), 391.37 (M + 1).

### 3.2 Photostability and Cytocompatibility Assessment of PNH

To test the photostability, PNH was continuously exposed to 10 W UV light for 12 h. PL spectra recorded after 4, 8, and 12 h of irradiation (Figure S5) revealed that both emission wavelength and intensity remain unchanged, demonstrating good photostability of the fluorophore. The biocompatibility of PNH was evaluated through MTT assay on HEK293T cells. As shown in Figure S6, PNH displayed a distinct dose-dependent increase in cell inhibition with an IC_50_ value of 11.22 ± 1.19 μM (logIC_50_ = 1.05 ± 0.075), corresponding to a 50% reduction in cell viability. At lower PNH concentrations, minimal cell inhibition was observed, indicating low cytotoxicity.

### 3.3 Fluorescence Features of PNH in Solution

#### 3.3.1 ICT Behavior

PNH displayed good solubility in DMF, DMSO, and THF, and the absorption spectra (Figure 1a) of these solutions showed gradual changes in the absorption wavelength (λ_max_) with increasing solvent polarity (THF < DMF < DMSO). PNH absorbed at 340 nm in THF, shifting to 370 nm in DMF and 375 nm in DMSO, indicating positive solvatochromism. Since the excited state of PNH is more polar than its ground state, polar solvents such as DMSO and DMF preferentially stabilize the excited state, thereby reducing the energy gap between the excited and ground states.^48–50^

**Figure 1:**
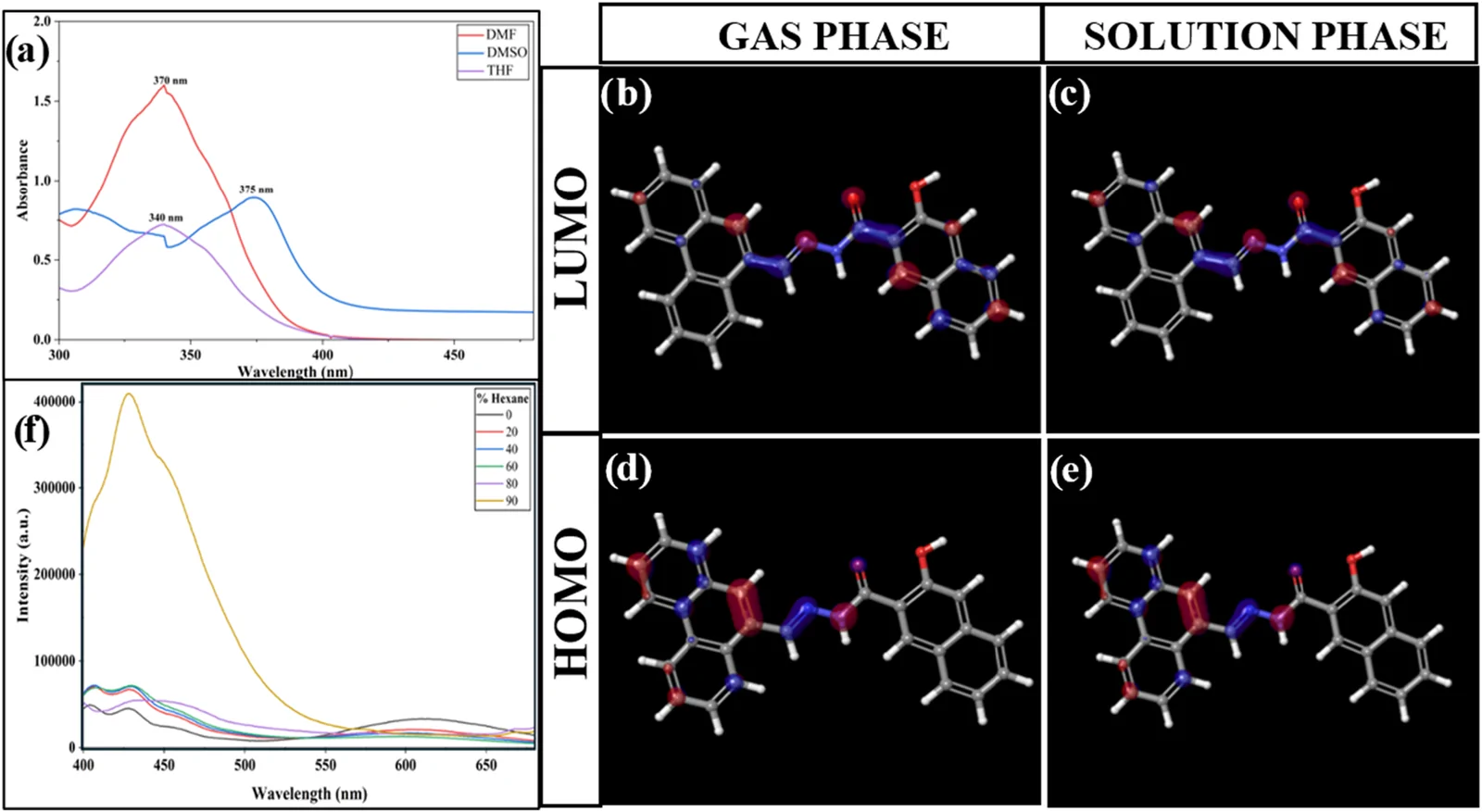
(a) Absorption spectra of PNH solutions (1 × 10^–5^ M). Molecular orbitals of single PNH: LUMO in (b) gas and (c) THF; and HOMO in (d) gas and (e) THF. (f) PL spectra of PNH in THF–hexane mixtures (1 × 10^–4^ M); λ_ex_ = 390 nm.

The geometry-optimized single, dimer, and trimer PNH atomistic snapshots, along with the π–π stacking in gas and solution phases are shown in Figures S7 and S8, respectively. The calculated molecular orbital energy values *E*_LUMO_ and *E*_HOMO_ and Δ*E* in the gas and solution phases are summarized in Table 3. The calculated LUMO–HOMO energy gap was ∼3.6 eV in both the phases. The LUMO and HOMO molecular orbitals of the optimized PNH molecule are presented in Figure 1b,1d for the gas phase and in Figure 1c,1e for the solution phase (THF), respectively. The ESP surface of PNH, along with the atomic notations in the gas and solution phases are shown in Figures S9 and S10, respectively. The atomic charges of PNH in the gas and solution phases are summarized in Tables S1 and S2, respectively. The atomic charges in the PNH reflect the electron density distribution within the molecule as obtained from electrostatic potential (ESP) calculations. The atomic charges of the carbonyl oxygen atom O19 (−0.47619), the phenolic oxygen atom O30 (−0.52874), and the phenolic carbon C22 (−0.59020) exhibited the highest negative atomic charges, indicating regions of high electron density in the gas phase. Slightly lower atomic charges of O19 (−0.55635), O30 (−0.58439), and C22 (−0.65965) were observed in the solution phase. The nitrogen atoms N16 (−0.22622) and N17 (−0.31283) were in the gas phase, while in the solution phase slightly lower values were observed for N16 (−0.30744), and in contrast, N17 (−0.26616) carried slightly lower negative charges owing to their lone-pair electrons. The carbonyl carbon C18 (0.67295) exhibited the highest positive charge, followed by C21 (0.50300) in the gas phase, reflecting their electron-deficient nature caused by the strong electron-withdrawing effect of the adjacent oxygen atoms. In the solution phase, C18 (0.67236) remained unchanged, while C21 (0.56077) exhibited slightly higher atomic charge. Among the hydrogen atoms, the hydroxyl proton H48 exhibited the highest positive charge, with values of 0.40639 in the gas phase and 0.44006 in the solution phase, indicating significant polarization of the O–H bond.

**Table 3:** Calculated Molecular Orbital Energy Values *E*_LUMO_ and *E*_HOMO_ and Δ*E* in Gas Phase and Solution Phase

PNH	gas-phase geometry optimization (Hartrees)	gas-phase geometry optimization (eV)	THF phase geometry optimization (Hartrees)	THF phase geometry optimization (eV)
*E*_LUMO_	–0.075237	–2.047302	–0.079670	–2.167930
*E*_HOMO_	–0.207333	–5.641817	–0.213370	–5.806092
Δ*E*	0.132096	3.594514	0.133700	3.638162

The ESP surfaces of PNH along with the atomic notations in the gas and solution phases are presented in Figures S9 and S10, respectively. The red regions correspond to negative ESP (high electron density), whereas the blue regions represent positive ESP (low electron density). Pronounced red regions are localized around the carbonyl oxygen (O19) and phenolic oxygen (O30) in both gas and solution phases, confirming these atoms as the most electron-rich sites in the molecule. Moderate negative potential is also observed around the nitrogen atoms (N16 and N17) in both gas and solution phases, reflecting their contribution to electron delocalization within the conjugated framework. Conversely, the blue region surrounding the hydroxyl hydrogen (H48) in both gas and solution phases indicates electron-deficient sites. The remaining aromatic rings predominantly exhibited white color regions, indicating relatively neutral electrostatic potential due to extensive π-electron delocalization. The DFT calculations on the single, dimer, and trimer PNH structures provide further insight into the relationship between the molecular structure and photophysical behavior of PNH. The frontier molecular orbital distributions and ESP demonstrate that the electronic density is delocalized over the conjugated framework, while intermolecular π–π interactions in the aggregated structures influence the electronic properties.

Further, to investigate the ICT behavior of PNH, its fluorescence properties were examined in THF–hexane mixtures with varying solvent compositions (Figure 1f). PNH exhibited relatively weak fluorescence emission in pure THF due to the stabilization of the ICT excited state in the more polar solvent. As the hexane fraction increased, the solvent polarity decreased, resulting in a progressive enhancement of the fluorescence intensity, which reached a maximum at 90% hexane. Furthermore, the CIE chromaticity diagram (Figure S11) revealed a solvent-dependent blue shift in the emission, corroborating the ICT characteristics of PNH. These observations indicate that the excited-state electronic distribution of PNH is highly sensitive to the surrounding dielectric environment, confirming the significant role of ICT in governing its photophysical behavior. Overall, the experimental and DFT results demonstrate that PNH exhibits pronounced ICT character, with its photophysical properties strongly governed by solvent polarity, molecular charge distribution, and intermolecular π–π interactions, thereby establishing its environment-sensitive optical response.

#### 3.3.2 AIE Behavior

PNH dissolves readily in THF but is insoluble in water. Aggregation was therefore triggered by gradually adding water to its THF solution. The AIE properties were investigated in THF–water mixtures, with water content varied from 0 to 99% while monitoring fluorescence changes. In pure THF, PNH showed weak emission. As the proportion of water increased from 0 to 60%, the fluorescence intensity steadily improved. A sharp rise in intensity is observed at 70% water, which peaks at 90%. However, at 99% water content, the emission intensity decreased, probably due to emission quenching by excessive aggregation. The PL spectra (Figure 2a) clearly demonstrate this progressive fluorescence enhancement with increasing water fraction. At 0% water, minimal emission indicates that the molecules remain dissolved, while the substantial increase in fluorescence at 90% water signifies the presence of AIE. Corresponding photographs (Figure 2b) captured under daylight and 390 nm UV illumination further illustrate these changes. The red shift suggests the formation of J-type aggregates featuring a head-to-tail stacking configuration, in contrast to H-aggregates that generally exhibit blue-shifted emission.^51–53^ SEM images support aggregation through the morphological observations. At 0% water content, small, well-dispersed crystals corresponding to monomeric species are observed (Figure 2c). In contrast, at 90% water content, larger, elongated, and densely packed structures are evident (Figure 2d), which indicates the presence of 1D or fibrous aggregates. In dilute solution, rotational and vibrational motions provide efficient nonradiative decay pathways, resulting in relatively weak emission. Upon aggregation, these molecular motions are restricted, suppressing nonradiative relaxation and enhancing radiative decay, thereby producing AIE. Furthermore, DFT calculations on the dimer and trimer structures suggest that intermolecular π–π interactions influence the electronic structure in the aggregated state.

**Figure 2:**
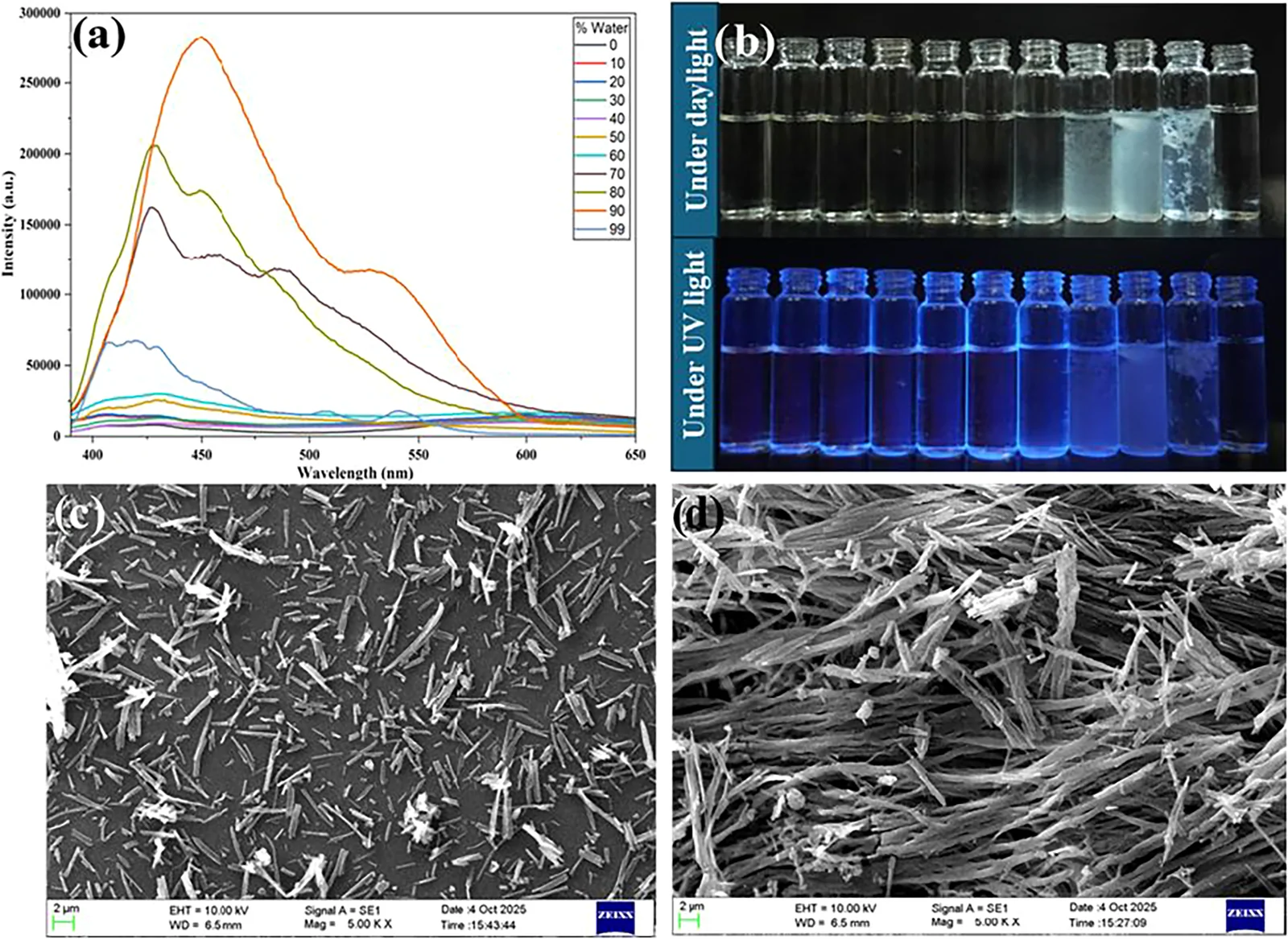
(a) PL spectra in THF–water mixtures (1 × 10^–4^ M); λ_ex_ = 390 nm. (b) Photographs of PNH in THF–water mixtures under daylight and 390 nm UV irradiation (0–99% water from left to right). SEM images of PNH in THF–water mixtures with (c) 0% and (d) 90% water fractions.

The particle size distribution graph for 0% water fraction (Figure S12a) showed a uniform spread, with the mean, median, and mode all measuring 161.1 nm. This suggests the particles are consistent in size. The cumulative distribution values are closely spaced (10% at 153.4 nm, 20% at 155.3 nm, and 40% at 159.2 nm), indicating good stability. No secondary peaks were observed, indicating that the system is monodisperse under anhydrous conditions. The particle size distribution graph for 90% water fraction (Figure S12b) showed significant aggregation in poor solvent conditions, with two peaks at 795 nm and 5.86 μm indicating polydispersity. The large *Z*-average diameter (2526.6 nm) and high polydispersity index (1.468) further confirm these observations. These results align with the AIE mechanism: at 0% water, particles remain stable and monodisperse on the nanoscale, while at 90% water, extensive aggregation occurs, forming large AIE-active particles.

### 3.4 Fluorescence Features of PNH Powder

#### 3.4.1 Acidofluorochromism

Acidofluorochromism refers to the reversible change in fluorescence properties of a substance in response to variations in pH.^54,55^ It is typically driven by structural alterations, such as protonation or deprotonation, that alter the absorption and emission spectra.^54,56–58^ Protonation-induced phase transitions can alter optical properties by affecting electron delocalization and energy levels.^59,60^ The acidofluorochromic behavior of PNH was studied through vapor-exposure experiments. Upon 10 min exposure to HCl vapors, white PNH turned yellow with greenish-yellow fluorescence under 390 nm UV light, while subsequent NH_3_ vapor treatment transformed it to pale-yellow with blue fluorescence. Reexposure to HCl restored the original color and fluorescence, demonstrating reversible and repeatable acid–base vapor responsiveness (Figure 3a). Consistent with these visual changes, pristine PNH displayed dual PL emission at 425 and 449 nm corresponding to blue fluorescence, which red-shifted to 453 and 532 nm, resulting in orange emission after HCl exposure. NH_3_ treatment restored the emission maxima to 427 and 454 nm, recovering the blue fluorescence (Figure 3b). The corresponding shifts in the CIE chromaticity diagram further confirmed the reversible acidofluorochromism (Figure 3c). The OH and NH groups are considered potential protonation/deprotonation sites due to the presence of available lone-pair electrons, and their acid–base-mediated electronic modulation may alter the conjugation and ICT character of PNH, resulting in the observed reversible fluorescence changes. Although the exact protonation site could not be experimentally established, the reversible spectral changes indicate modulation of the electronic structure under acidic and basic conditions.

**Figure 3:**
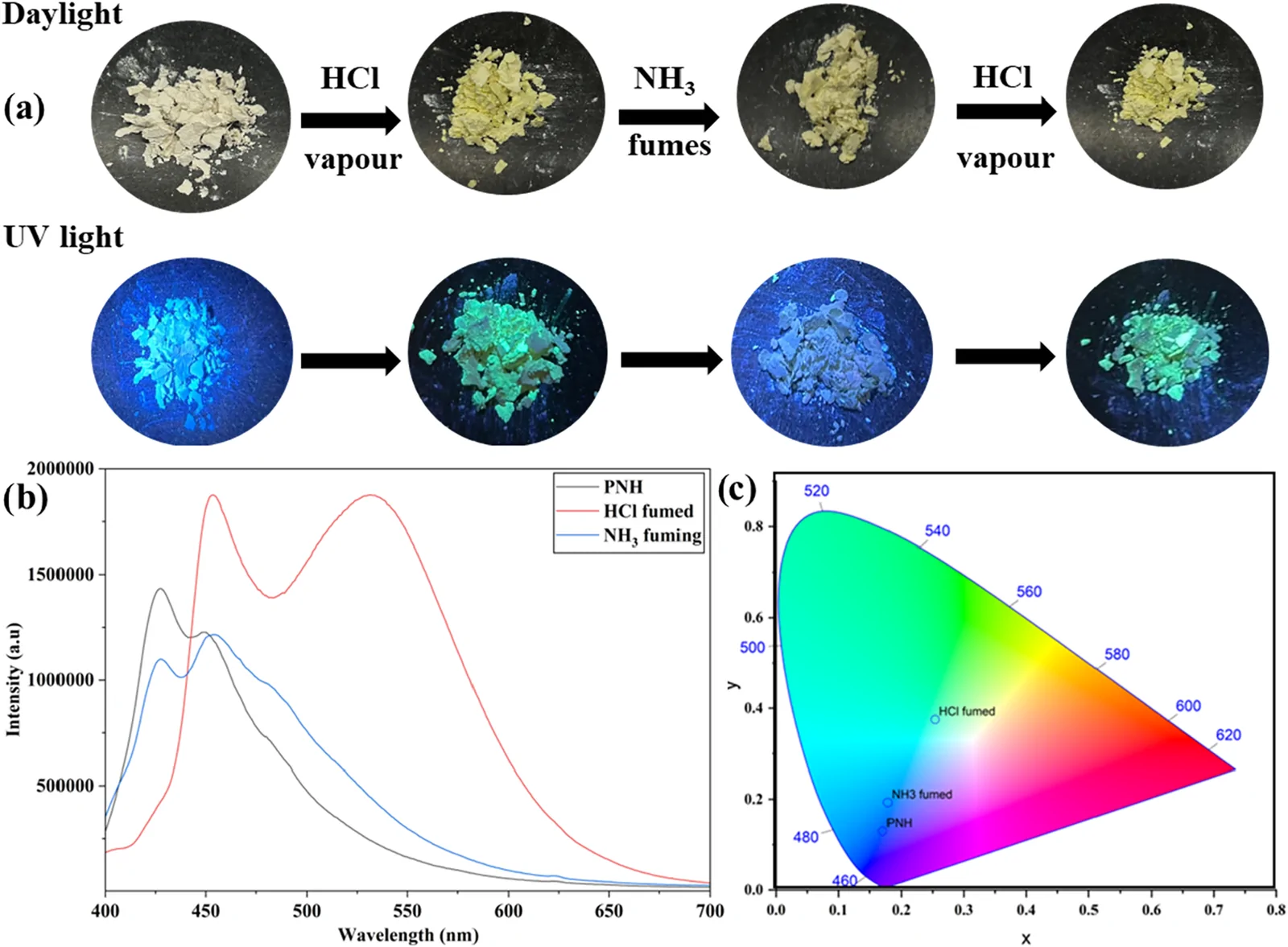
(a) Photographs illustrating acidofluorochromic behavior of PNH, (b) emission spectra of PNH powder fumed with HCl and NH_3_, and (c) corresponding chromaticity plot; λ_ex_ = 390 nm.

To gain further insight into the acid-induced fluorescence switching, FTIR spectra of pristine and HCl-fumed PNH were compared (Figure S13). The HCl-fumed sample exhibited slight changes in the broad O–H/N–H stretching region (3200–3600 cm^–1^) and minor variations in the fingerprint region, indicating perturbation of the hydrogen-bonding environment and local electronic structure upon acid exposure. However, no significant shift in the characteristic vibrational bands was observed. Therefore, the reversible acidofluorochromic behavior is attributed to the reversible modulation of the electronic structure upon acid/base treatment.

#### 3.4.2 Mechanofluorochromism

The effect of mechanical stress on the fluorescence response of PNH was studied by grinding the pristine sample with a mortar and pestle. Significant changes were observed under daylight and 390 nm UV illumination, with the fluorescence changing from blue to orange upon grinding (PNH-g). This response was reversible as the DCM vapor restored the orange fluorescence to blue. PL measurements at 390 nm further confirmed this behavior: pristine PNH exhibited peaks at 430 and 450 nm, which shifted to 440 and 560 nm after grinding, corresponding to an orange emission. Upon DCM vapor exposure, the peaks of PNH-*g*-DCM recovered to 430 and 452 nm (Figure 4a), confirming the reversible mechanofluorochromic response.

**Figure 4:**
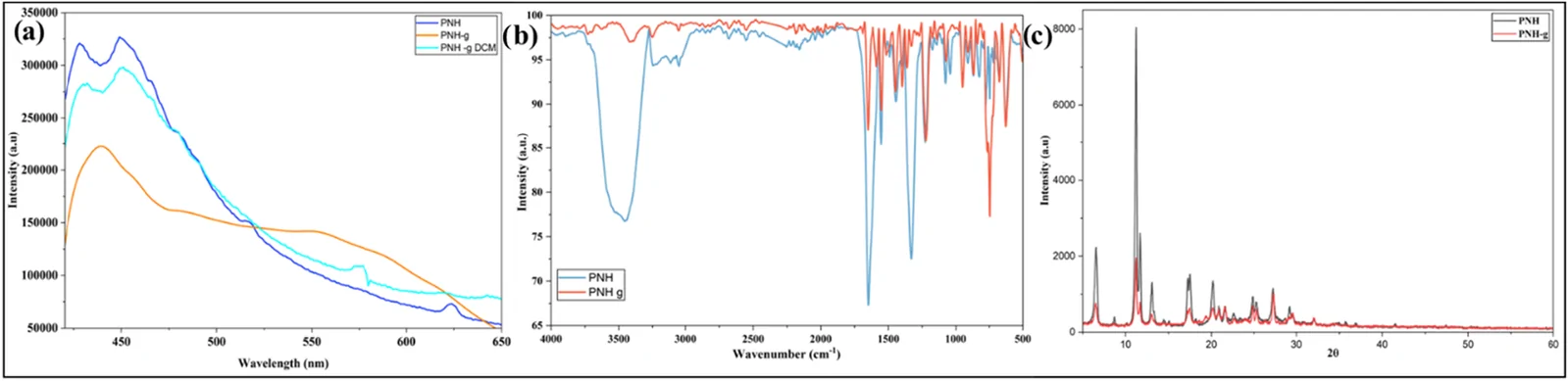
(a) Emission spectra of PNH and PNH-g; λ_ex_ = 390 nm, (b) FTIR, and (c) XRD plots of PNH and PNH-g.

The comparative quantitative FTIR analysis of PNH and PNH-g (Figure 4b) demonstrated distinct spectral variations, indicating grinding-induced changes in molecular packing and intermolecular interactions. The broad N–H/O–H stretching band at 3400–3600 cm^–1^ became weaker and narrower in PNH-g, suggesting partial disruption of intermolecular hydrogen bonding. Noticeable shifts and intensity changes in the fingerprint region (1600–800 cm^–1^), particularly for aromatic C=C, C=N, and C–N stretching modes, further indicated altered molecular packing and weakened supramolecular interactions. These changes suggest that mechanical stress primarily modifies hydrogen bonding and molecular packing rather than the chemical structure, thereby altering the optical properties and producing the observed mechanofluorochromic response.

The sharp PXRD reflections of pristine PNH confirm its crystalline nature, whereas grinding markedly reduced the diffraction intensity in PNH-g (Figure 4c), indicating decreased crystallinity and increased structural disorder without altering the fundamental crystal phase.^11^ This is consistent with the reduction in the crystallite size from 36.97 to 30.59 nm. Such grinding-induced loss of crystallinity, commonly associated with disruption of intra/intermolecular hydrogen bonding, metastable-phase formation, and partial amorphization, alters molecular packing and fluorescence behavior.^61–64^ SEM imaging further supports this structural disruption: pristine PNH (Figure S14a) exhibited densely packed, well-defined elongated rod/needle-like microcrystals with sharp edges and uniform surfaces, whereas PNH-g (Figure S14b) showed smaller, irregularly fragmented, rough, and agglomerated particles with fractured crystal structures. Collectively, these results indicate that the mechanofluorochromic response arises from grinding-induced modifications in molecular packing and intermolecular π–π interactions, which alter the excited-state electronic structure and consequently the fluorescence emission.

To quantitatively investigate the mechanofluorochromic behavior of PNH, pellets were prepared under nominal pressures of 0.018–0.088 MPa. Representative photographs and normalized PL spectra are provided in Figures S15 and S16, respectively. The spectra were normalized to their maximum emission intensity to compare the pressure-dependent changes. At 0.018–0.035 MPa, the emission remained similar to that of pristine PNH, with maxima at 430–450 nm. Increasing the pressure to 0.053–0.071 MPa produced a progressive red shift toward 475–500 nm, indicating pressure-induced changes in molecular packing and intermolecular interactions. The most pronounced spectral change occurred between 0.035 and 0.053 MPa, suggesting that the mechanofluorochromic transition becomes significant within this range. At 0.088 MPa, the emission broadened with a reduced spectral resolution, indicating further modification of the emissive state. The reported pressures are nominal, as the pressure distribution across the pellet surface is not perfectly uniform; nevertheless, the results provide a quantitative assessment of the pressure-dependent mechanofluorochromic response of PNH.

Overall, the multifunctional behavior of PNH originates from the synergistic relationship between its donor–acceptor molecular structure, extended π-conjugated framework, and intermolecular interactions. The ICT character governs the solvatochromic and acidofluorochromic responses, whereas restriction of intramolecular motion in the aggregated state produces aggregation-induced emission. Mechanical stimulation modifies molecular packing and intermolecular π–π interactions, giving rise to reversible mechanofluorochromism. These complementary structure–property relationships collectively enable the multifunctional performance of PNH in anticounterfeiting and forensic applications. Although the proposed mechanisms are supported by complementary experimental and computational evidence, direct crystallographic confirmation of molecular packing changes by single-crystal X-ray diffraction could not be achieved because suitable single crystals were not obtained despite extensive crystallization attempts. Furthermore, unambiguous identification of the protonation site requires additional investigation. These aspects remain important directions for future work.

### 3.5 Print Properties of PNH Screen Ink

The colorimetry and optical density study results (Table 4) show that the water-based eco-friendly PNH ink exhibits good printability, mechanical durability, and photostability. Unprinted paper showed a high L* value of 92.24 and low optical density (0.1), indicating high brightness and no ink deposition. After printing, L* decreased to 89.07, showing reduced brightness due to ink film deposit, while a* and b* increased from 0.53 to 2.60 and 7.68 to 14.88, respectively, indicating a distinct shift toward a yellowish-red hue. The Δ*E* of 8.4 confirmed a perceptible color difference, while the density increased to 0.3, demonstrating effective ink transfer and uniform film formation.

**Table 4:** Colorimetric and Density Data of Blank and Printed Paper Samples before and after UV Exposure and Rub Tests

paper sample	L*	a*	b*	Δ*E*	density
unprinted	92.24	0.53	7.68		0.1
printed	89.07	2.6	14.88	8.4	0.3
photostability test (different cycles of UV exposure)					
print unexposed to UV	89.07	2.6	14.88		0.3
print exposed to 10 cycles	89.02	2.4	14.67	0.29	0.3
print exposed to 20 cycles	88.15	1.77	14.11	1.46	0.3
print exposed to 30 cycles	87.8	1.72	13.86	1.85	0.3
print exposed to 40 cycles	87.54	1.68	13.8	2.09	0.3
print exposed to 50 cycles	87.16	1.59	13.78	2.42	0.3
rub resistance test (different number of rubs)					
unrubbed print	89.07	2.6	14.88		0.3
print after 25 rubs	88.8	1.93	13.57	1.50	0.4
print after 50 rubs	88.15	1.77	14.11	1.46	0.4
print after 75 rubs	85.4	3	16.03	3.87	0.4
print after 100 rubs	85.2	2.2	16.12	4.08	0.4

Photostability was assessed over 50 repeated UV-exposure cycles. L* gradually decreased from 89.02 (10 cycles) to 87.16 (50 cycles), while a* and b* decreased from 2.40 to 1.59 and 14.67 to 13.78, respectively, indicating a slight shift toward greenish-yellow. The Δ*E* increased from 0.29 to 2.42, representing minimal, visually acceptable color variation within security-printing tolerance limits. Notably, the optical density remained constant at 0.3 throughout all cycles, confirming excellent photostability without significant pigment degradation or material loss.

The mechanical durability of the printed ink was further evaluated using the rub resistance test. After 25 and 50 rubbing cycles, only minimal changes were observed, with Δ*E* values of 1.50 and 1.46, respectively, indicating negligible visual color change. At higher abrasion levels (75 and 100 rubs), the L* value decreased more noticeably to 85.40 and 85.20, while the b* value increased to 16.03 and 16.12, indicating a slight enhancement in yellow tone. Correspondingly, the Δ*E* values increased to 3.87 and 4.08, representing moderate but still acceptable visual changes for printed security features. Notably, the optical density increased and stabilized at 0.4 after rubbing, confirming strong ink adhesion and resistance to mechanical wear without film delamination. The colorimetric plots for unprinted blank, printed, and print proofs exposed to UV and rub test are depicted graphically in Figure S17.

### 3.6 Security Features of PNH Screen Print

The PNH-inked paper exhibited a clear mechanofluorochromic sensitivity. Under daylight, the printed area appeared white and showed bright blue fluorescence under 390 nm UV illumination, which changed to orange upon mechanical scratching or rubbing, as demonstrated in Supporting Video 1. DCM vapor exposure restored the original fluorescence, confirming the reversibility. Images of the screen-printed sample under daylight, UV illumination, and after rubbing are shown in Figure 5a, with corresponding emission spectra in Figure 5b. Under 390 nm excitation, the pristine print exhibited an emission maximum at 428 nm, whereas the rubbed print (RP) showed a red-shifted, broadened profile with major bands around 400 and 510 nm, attributed to mechanically induced molecular rearrangement. DCM fuming (RP-DCM) restored the original emission, confirming the reversible mechanofluorochromism. These results closely match the solid-state behavior of PNH, demonstrating that its fluorescence switching is retained in the ink and after being printed onto surfaces.

**Figure 5:**
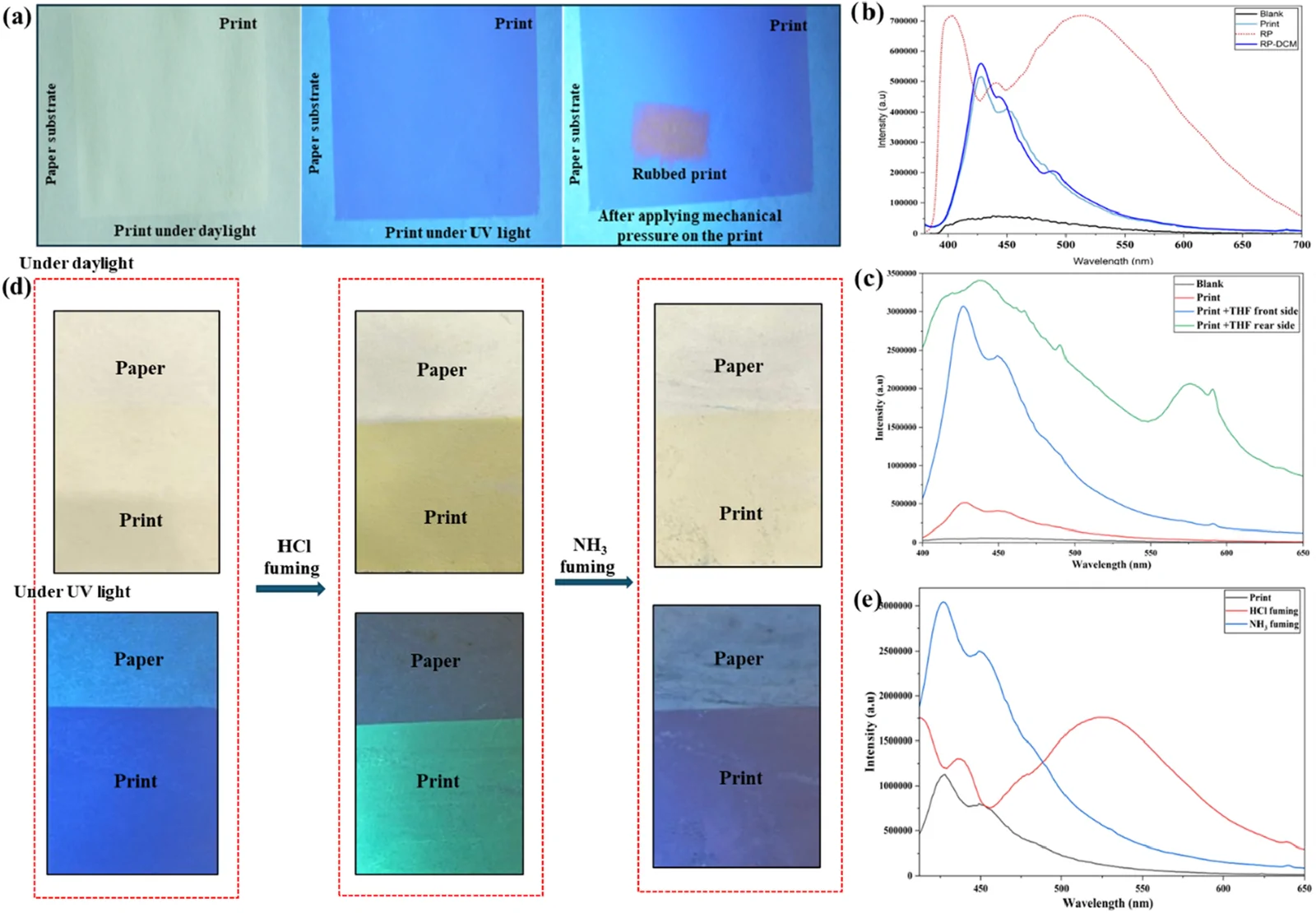
(a) Photographs of screen print under daylight, UV light, and the rubbed print exposed to UV light. (b) Comparison of emission spectra for unprinted, printed, RP, and RP-DCM samples. (c) Emission spectra of print and THF-treated print: front and rear sides. (d) Photographs of untreated, HCl-fumed, and NH_3_-fumed prints under daylight and UV light. (e) Emission spectra of print, HCl-fumed, and NH_3_-fumed prints; λ_ex_ = 390 nm.

A pronounced orange-red emission was observed on the rear side of the THF-exposed printed region (Figure S18b), whereas no corresponding fluorescence appeared on the front side (Figure S18a). PL spectra of the THF-treated front and rear sides (Figure 5c) confirmed a new emission peak at 575 nm on the rear side, providing an additional authentication feature. The acidofluorochromic response was also demonstrated in screen prints: after 10 min of HCl exposure, the print showed greenish-yellow fluorescence under UV light, which reverted to blue upon NH_3_ fuming. These reversible changes are shown visually and spectroscopically in Figure 5d,5e, with emission shifting to 525 nm after HCl treatment and returning to 428 nm after NH_3_ exposure; the chromaticity plot (Figure S19) further confirms the reversible switching. Overall, water-based PNH ink provides multiple security features, including daylight invisibility, reversible mechanofluorochromism, acid–base vapor responsiveness, and THF-induced rear-side fluorescence.

### 3.7 Cycling Stability of Reversible Fluorochromic Behavior

The long-term durability of PNH-based printed security patterns was evaluated through 50 consecutive acidofluorochromic and mechanofluorochromic switching cycles. For the acidofluorochromic response, repeated HCl/NH_3_ vapor cycles produced only a gradual decrease in fluorescence intensity (Figure S20a). After 50 cycles, HCl-treated and NH_3_-recovered prints retained 91.7 and 93.0% of their initial fluorescence intensities, respectively. The reversible transition between protonated and deprotonated states remained clearly distinguishable throughout the cycling process, confirming an excellent reversibility. The slight intensity loss may arise from minor irreversible changes in molecular packing or the printed film caused by repeated vapor exposure and protonation–deprotonation processes. The corresponding emission spectra are shown in Figure S20b. Overall, fluorescence retention above 91% after 50 cycles demonstrates excellent operational stability and durability, supporting the potential of PNH prints for reusable anticounterfeiting applications.

Mechanofluorochromic stability was assessed through 50 consecutive rubbing/solvent recovery cycles using mechanical rubbing followed by DCM vapor treatment. As shown in Figure S21a, fluorescence intensity remained nearly unchanged during the first 20 cycles, followed by a gradual decrease. After 50 cycles, rubbed and DCM-treated prints retained 93.4 and 91.7% of their initial fluorescence intensities, respectively. Despite this slight decrease, the mechanically induced and DCM-recovered fluorescence states remained clearly distinguishable throughout the cycling process, confirming the excellent durability and reusability of the printed security labels. The corresponding emission spectra are presented in Figure S21b.

### 3.8 PNH as a Forensic Tool for LFP and LLP Development and Visualization

Fluorophores have emerged as effective tools for developing and visualizing latent finger and lip marks because of their high sensitivity and strong emission contrasts under UV illumination. They interact with various constituents of latent prints such as amino acids, fatty acids, lipids, sweat residues, and sebaceous secretions through hydrogen bonding, hydrophobic interactions, π–π stacking, and electrostatic forces. These selective bindings enhance fluorescence intensity at the print ridges while reducing background interference, enabling clear visualization and capturing of high-resolution images. The forensic efficiency of a fluorophore depends on its affinity for biological components and its ability to generate stable, high-contrast luminous patterns. Accordingly, PNH was investigated for its interactions with glycine, the most prevalent fingerprint amino acid, and myristic acid, the primary lipid, followed by visualization of fingerprints and lip prints deposited on various surfaces using PNH.

#### 3.8.1 Interaction Studies Using MD Simulation

##### 3.8.1.1 Hydrogen-Bond Interactions

The dynamic interactions of PNH molecules in the hydrophilic and hydrophobic surface types were investigated via hydrogen-bond and interaction energy analyses. Representative snapshots of the surface from MD simulations show a hydrophilic surface in Figure 6a and hydrophobic surface in Figure 6b. As PNH molecules are hydrophobic, they show preferential adsorption and lie close to the hydrophobic surface as shown in Figure 6b. To investigate the detailed inter- and intramolecular interactions, the hydrogen-bonding networks (shown in yellow dashed lines, nonbonded interactions) were analyzed. Interactions were identified between myristic acid–glycine (Figure 6c) and PNH–glycine (Figure 6d). Hydrogen bonds formed between the nitrogen-bound hydrogen of glycine and the carboxyl oxygen of myristic acid, as depicted in Figure 6c. Further, the hydrogen-bonded interaction between the nitrogen-bound hydrogen atom of glycine and the oxygen atom of the hydroxyl group in PNH is illustrated in Figure 6d. The number of hydrogen bonds in pairs are summarized in Table 5.

**Figure 6:**
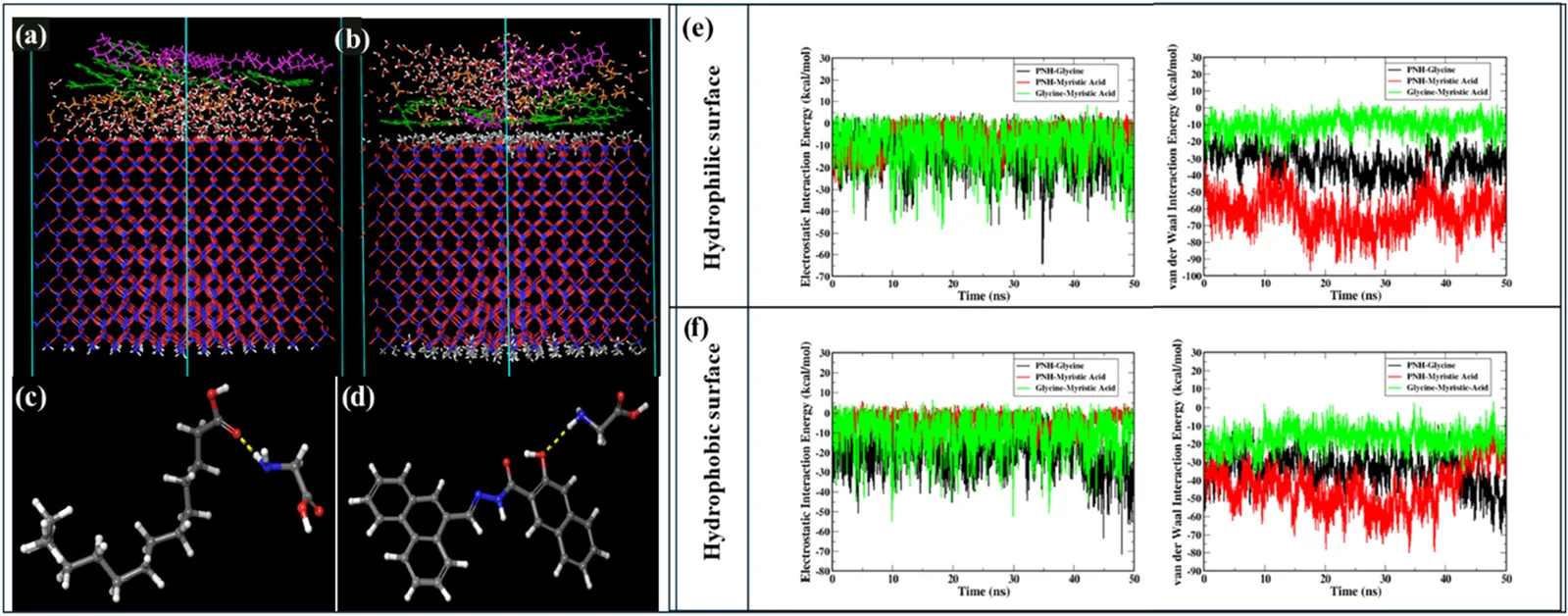
(a) Hydrogen-terminated SiO_2_(001) surface: hydrophilic and (b) methyl-terminated SiO_2_(001) surface: hydrophobic (color coding: glycine in orange, PNH in green, myristic acid in pink, silicon in blue, oxygen in red, hydrogen in white, and methyl group carbon in gray in both models). Hydrogen bond between (c) glycine and myristic acid and (d) PNH and glycine (color coding: nitrogen in blue, oxygen in red, hydrogen in white, and carbon in gray in both panels (c) and (d)). Electrostatic and van der Waals interaction energy versus simulation time for different pairs on (e) hydrophilic and (f) hydrophobic surfaces.

**Table 5:** Number of Hydrogen Bonds on Different Surfaces

sl. no.	pairs	hydrophilic surface	hydrophobic surface
1	PNH–glycine	4.428	4.289
2	glycine–myristic acid	5.429	5.209

##### 3.8.1.2 Interaction Energy Analysis

Pairwise interaction energies were calculated between PNH–glycine, PNH–myristic acid, and glycine–myristic acid on the hydrophilic and hydrophobic surfaces and corresponding interactions are presented in Supporting Videos 2 and 3. Energy values for the hydrophilic and hydrophobic surfaces are compiled in Table 6. Van der Waals energies are dominant over electrostatic energies on both surfaces. PNH–myristic acid van der Waals interaction energies are higher compared to all other pairs. Figure 6e,f portrays the interaction energy analysis along hydrophilic and hydrophobic surfaces versus simulation time. Interestingly, the PNH–myristic acid pair shows the lowest total interaction energy of −68.97 kCal/mol compared to PNH–glycine and glycine–myristic acid on a hydrophilic surface. However, PNH–glycine has the lowest total interaction energy of −4.61 kCal/mol among PNH–myristic acid and glycine–myristic acid on a hydrophobic surface.

**Table 6:** Interaction Energies: Hydrophilic and Hydrophobic Surfaces

no.	pairs	electrostatic energy (kcal/mol)	van der Waals energy (kcal/mol)	total interaction energy (kcal/mol)
hydrophilic surface				
1	PNH–glycine	–14.4081	–32.6584	–47.0665
2	PNH–myristic acid	–6.4644	–62.5101	–68.9745
3	glycine–myristic acid	–9.7377	–10.5592	–20.2969
hydrophobic surface				
1	PNH–glycine	–19.6253	–34.9913	–54.6166
2	PNH–myristic acid	–4.1232	–44.5419	–48.6651
3	glycine–myristic acid	–10.3496	–17.1677	–27.5173

#### 3.8.2 FTIR Analysis of Molecular-Interaction Dynamics

FTIR spectral analysis was used to study intermolecular interactions among glycine, myristic acid, water, and PNH, as well as supramolecular associations in binary and multicomponent systems (Figure S22a). Figure S22b presents a magnified view of the designated spectral region depicted in Figure S22a, thereby enhancing clarity and facilitating a more detailed observation of the changes detected. Glycine (G) exhibits a broad band between 3000 and 2800 cm^–1^, attributed to hydrogen-bonded N–H stretching, along with characteristic carboxylate stretching bands at 1578 cm^–1^ (asymmetric COO^–^) and 1390 cm^–1^ (symmetric COO^–^), confirming its zwitterionic nature. Upon addition of water (G+W), the stretching region became wider and more intense, indicating enhanced hydrogen-bonding interactions between water molecules and the polar functional groups of glycine. Myristic acid (MA) shows characteristic absorption bands at 2920 and 2850 cm^–1^, corresponding to asymmetric and symmetric C–H stretching vibrations of the alkyl chain, along with a sharp band at 1696 cm^–1^ assigned to carboxylic acid C=O stretching. Bands in the 1460–1400 cm^–1^ region are attributed to CH_2_ bending, while the peak at 730 cm^–1^ corresponds to methylene rocking, confirming the long-chain aliphatic nature of MA. In the MA+W system, the emergence of a broad band in the 3200–3500 cm^–1^ region indicates strong −OH stretching associated with hydrogen bonding between water molecules and the MA carboxylic headgroup, accompanied by variations in the carbonyl band intensity and shape, confirming interaction at the polar −COOH site (Figure S22c and an enlarged region shown in Figure S22d). PNH displays a broad absorption band in the 3200–3500 cm^–1^ region due to overlapping phenolic O–H and hydrazide –NH stretching vibrations, along with a prominent C=O stretching band at 1640–1642 cm^–1^. In the PNH+G system, increased broadening in the hydroxyl and amine stretching region and noticeable changes in the carbonyl and carboxylate regions confirm strong intermolecular hydrogen-bonding interactions between PNH (−OH, -NH, C=O) and glycine (NH_3_^+^/COO^–^). Similarly, the MA+W+PNH system exhibits significant broadening of the O–H/N–H stretching region and modification of the carbonyl band with a shift to ∼1638 cm^–1^, indicating strong hydrogen bonding between myristic acid and PNH, with water as the bridging medium.

Overall, the comparative FTIR spectra indicate that the multicomponent system is dominated by noncovalent supramolecular interactions, especially hydrogen bonding and electrostatic association, with no evidence of covalent bond formation. The final mixture (G+MA+W+PNH) as shown in Figure S22e displays maximum peak broadening and intensity changes in O–H/N–H and carbonyl/carboxylate regions, confirming an extensive hydrogen-bonded network. Figure S22f shows clear peak shifts, which strongly support the proposed mechanism wherein PNH effectively interacts with both hydrophilic (glycine/water) and lipid-rich amphiphilic (myristic acid) environments, improving stabilization, adhesion, and molecular retention within the fingerprint-residue components.

#### 3.8.3 PNH Enabled LFP Development and Visualization

LFPs are crucial forensic evidence in criminal investigations.^65–70^ LLPs provide a complementary biometric tool, as their distinct and stable patterns can identify individuals, including identical twins, although surgery or trauma may alter these features.^71–75^ The development and visualization of latent marks increase the ability to link suspects to crime scenes.^69,76^

##### 3.8.3.1 LFP Visualization on Nonporous Surfaces

PNH powder produced a distinct fingerprint image on nonporous glass under 390 nm UV light (Figure 7a). The rectangular area in Figure 7a was analyzed to obtain the grayscale pixel profile and the 3D interactive plot. The grayscale pixel profile (Figure 7b) clearly distinguished ridges and furrows, providing enhanced contrast and pattern visualization. The 3D interactive profile (Figure 7c) distinctly displayed ridge–valley patterns, depth variations, and surface topography through color gradient, enabling detailed forensic analysis. The print showed distinct level 1 loop patterns, level 2 ridge endings and bifurcations, and clearly visible level 3 sweat pores (highlighted in Figure 7d).

**Figure 7:**
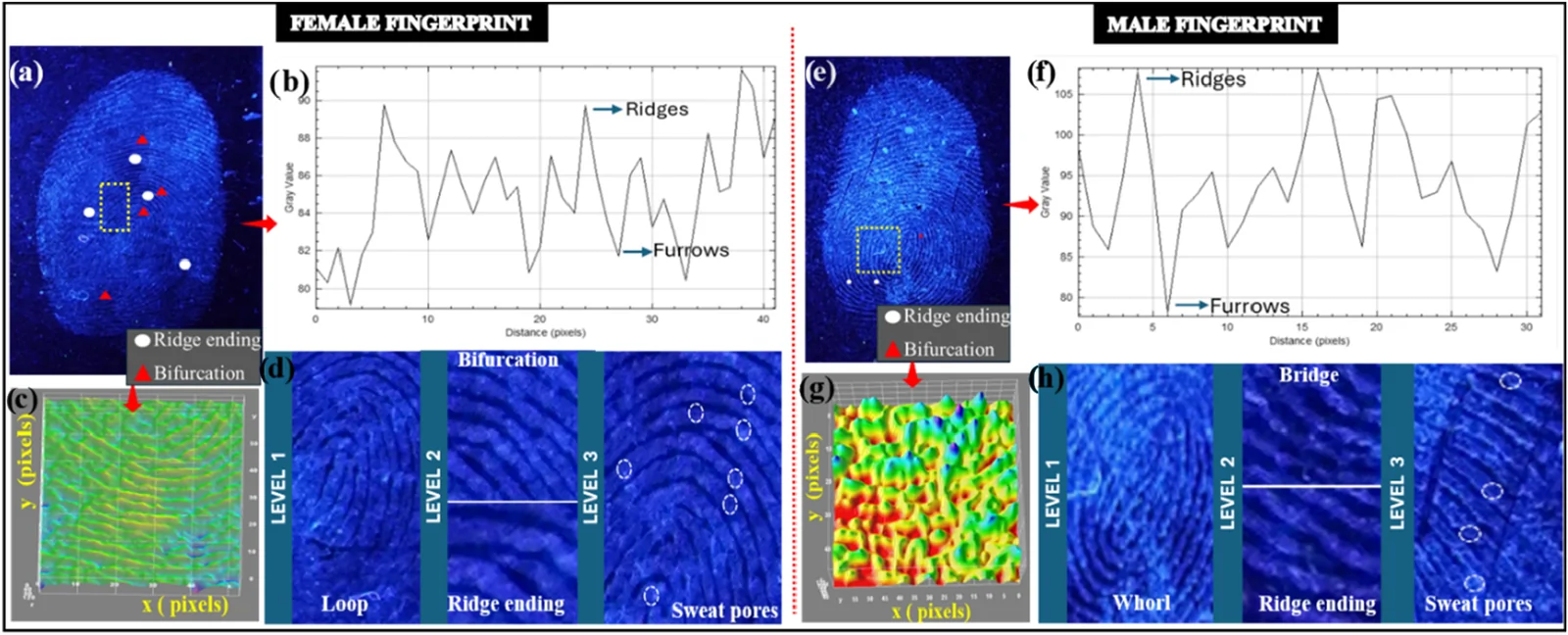
(a) Photograph, (b) grayscale pixel profile, (c) 3D interactive plot, and (d) level 1–3 features of LFP from the female donor developed on a nonporous glass surface. (e) Photograph, (f) grayscale pixel profile, (g) 3D interactive plot, and (h) level 1–3 features of LFP from the male donor developed on a nonporous glass surface.

##### 3.8.3.2 Gender Differentiation Using PNH-Dusted Fingerprints

Although fingerprints are inherently unique to individuals, gender-related differences in ridge and pore morphology have been explored for forensic anthropology and biometric gender estimation.^77,78^ Females generally exhibit finer, denser ridges, with ≥12 ridges per 25 mm^2^ typically indicating female and ≤11 ridges per 25 mm^2^ suggesting male fingerprints,^79,80^ although the average differences are small but statistically significant.^81^ Other reported features include sweat-pore distribution and morphology,^82,83^ ridge thickness,^77^ and fingerprint patterns such as loops, whorls, and arches.^78^ However, variations among studies and methodologies, along with demographic performance gaps in fingerprint recognition systems,^84^ highlight the need for standardized protocols and larger, diverse data sets. Nevertheless, ridge- and pore-based gender estimation models have achieved accuracies exceeding 96% in some studies.^85^

In the present study, PNH was used to develop LFPs from male and female donors (Figure 7a,7e). Comparative grayscale pixel profiles (Figure 7b,7f) and 3D interactive plots (Figure 7c,g) revealed lower ridge density, wider ridges, and greater pore spacing in male fingerprints than in female fingerprints. Female prints showed higher sweat-pore density due to narrower ridges and closer pore spacing (Figure 7d,7h). Level 2 features were also identified for both genders, with white circles marking ridge endings and red triangles indicating bifurcations in Figure 7a,7e.

##### 3.8.3.3 LFP Visualization on Semiporous Surfaces

To further evaluate its effectiveness, PNH was applied to semiporous glossy paper, resulting in distinct LFP visualization (Figure 8a). Both grayscale pixel intensity analysis (Figure 8b) and 3D profiling (Figure 8c) revealed a strong ridge–furrow contrast. The fingerprint showed level 1 loop patterns and level 2 features like bridges, ridge dots, bifurcations, and short ridges, along with sweat pores (marked by white circles in Figure 8d). The findings demonstrate that PNH is an efficient and dependable material for LFP visualization on both nonporous and semiporous surfaces under UV light.

**Figure 8:**
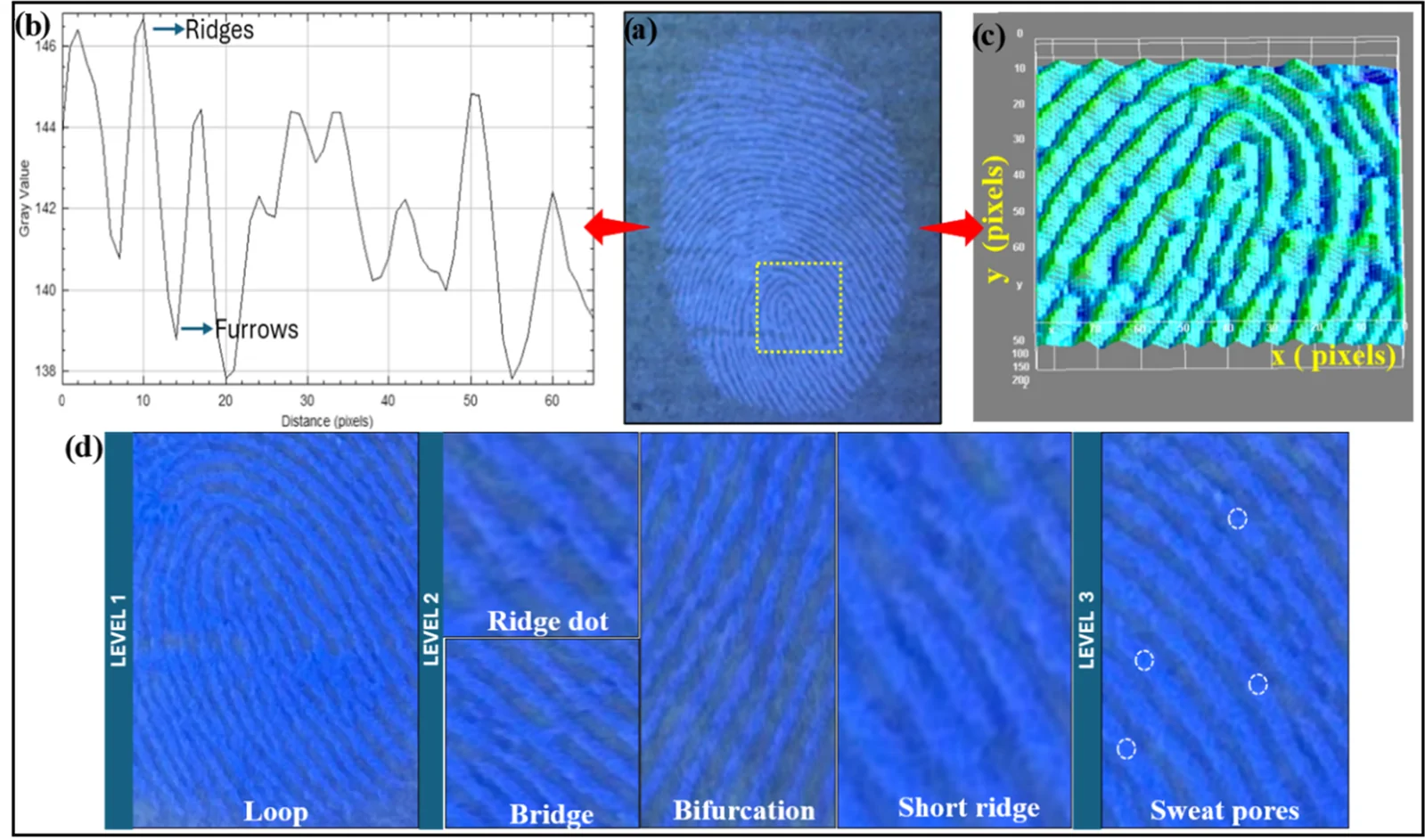
(a) Photograph, (b) grayscale pixel profile, (c) 3D interactive plot, and (d) level 1–3 features of LFP developed on semiporous glossy surfaces.

##### 3.8.3.4 LFP Visualization on Challenging Surfaces

Developing LFPs on challenging surfaces remains a major concern due to poor residue retention and low contrast. Figure 9a shows LFP developed on a reflective CD surface, while Figure 9b offers a closer view. The highlighted region in Figure 9a analyzed by grayscale pixel profiling (Figure 9c) showed clear ridge–furrow contrast, and 3D plot (Figure 9d) demonstrated depth changes in ridge–valley pattern. The prints displayed distinct ridge patterns with level 1–3 features (Figure 9e). Yet another key challenge in fingerprint visualization is the same-background effect, particularly on light-colored substrates. Since PNH is white, its performance was evaluated on a white tile. Despite negligible contrast under daylight conditions (Figure 9f), the fingerprint became distinct under UV illumination (Figure 9g). Grayscale pixel intensity analysis of the highlighted region (Figure 9i) clearly differentiated ridges and furrows, while 3D interactive surface profiling (Figure 9h) enhanced contrast and depth perception, revealing ridge flow and ridge–valley morphology. Well-defined level 1–3 features were clearly identified (Figure 9j), confirming the effectiveness of PNH even on substrates with a matching background color.

**Figure 9:**
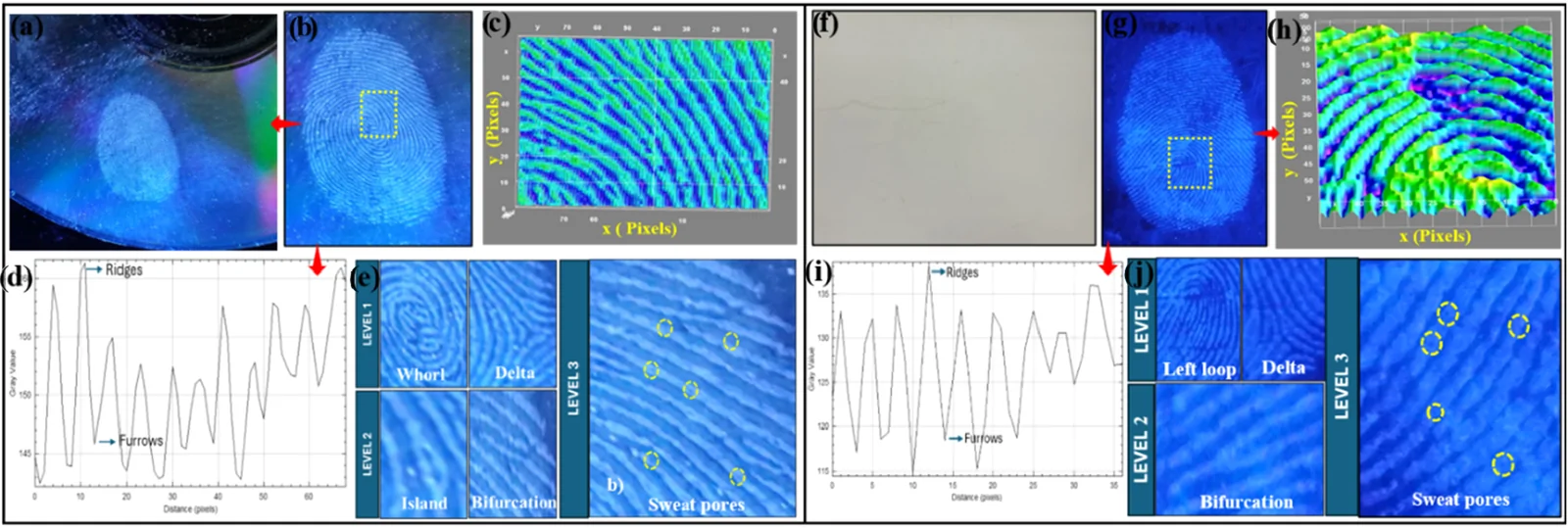
(a) Photograph of LFP developed on the nonporous CD surface, (b) its magnified image under UV light, (c) 3D interactive plot, (d) grayscale pixel profile, and (e) level 1–3 features. Photographs of LFP developed under (f) daylight and (g) 380 nm UV light on white tile, (h) 3D interactive plot of the selected region, (i) grayscale pixel profile, and (j) level 1–3 features.

To further evaluate the practical forensic applicability of PNH, its fingerprint development performance was compared with that of a commercially available fingerprint-developing powder under identical conditions (Figure S23). While both materials successfully visualized the LFP pattern, PNH produced brighter fluorescence and superior ridge–furrow contrast under UV illumination. The grayscale intensity profile of the PNH-developed fingerprint exhibited larger intensity differences between ridge and furrow regions than those of the commercial powder, indicating enhanced image contrast and improved discrimination of fingerprint features. These results demonstrate that PNH provides more effective fingerprint visualization than does the commercial powder under the investigated conditions.

#### 3.8.4 Influence of Substrate Properties on Fingerprint Development

To understand substrate-dependent fingerprint development, static water contact angles were measured for representative substrates, following the order CD (90.03 ± 4.21°) > paper (69.98 ± 5.11°) > glass (60.86 ± 8.97°) > tile (46.07 ± 1.81°), indicating distinct surface wettability. Surface profilometry of CD showed a relatively smooth surface with average roughness (Ra) = 0.2565 μm, root-mean-square roughness (Rq) = 0.3888 μm, maximum profile height (Rz) = 1.678 μm, total profile height (Rt) = 6.544 μm, and isotropy = 35.02%. The 3D profile of the CD-developed LFP is shown in Figure S24. Substrate wettability, roughness, porosity, and surface chemistry collectively influence the spreading and retention of LFP residues containing hydrophilic eccrine sweat and hydrophobic sebaceous lipids. On the hydrophobic, smooth CD, localized lipid-rich residues promote PNH adsorption, yielding high fluorescence contrast and well-defined ridges. More wettable ceramic tiles facilitate greater spreading of aqueous residues, slightly reducing ridge sharpness. Porous paper partially absorbs aqueous components while providing anchoring sites for PNH, enhancing ridge retention, whereas smooth, nonporous glass retains residues mainly at the surface, allowing selective PNH adherence to ridge deposits. These observations agree with our previous studies using ATH and BTAF fluorescent powders, where substrate topography and roughness influenced powder distribution, residue retention, and ridge clarity.^9,86^ Collectively, PNH visualization is governed by the interplay of substrate wettability, roughness, porosity, and surface chemistry; nevertheless, it consistently produced clear, high-contrast ridge patterns across all tested substrates. Its strong solid-state fluorescence and favorable interactions with fingerprint residues enable effective LFP development across forensic surfaces with diverse surface properties.

### 3.9 PNH Enabled Latent Lip-Print Development and Visualization

The permanent patterns of lip prints collected at crime scenes enable personal identification in forensic cases. Many scientists including Suzuki and Tsuchihashi, Martin Santos, Renaud, Afchar Bayar, and Jose Maria Dominguez had devised different classification systems based on the shape of patterns across lips.^87–91^

#### 3.9.1 LLP Visualization on Nonporous Surfaces

Figure 10a shows an LLP developed with PNH on glass under 390 nm UV illumination. The grayscale pixel intensity profile (Figure 10b) highlights tonal variations, with darker regions representing denser, prominent features and lighter regions indicating less-defined features. The corresponding 3D interactive profile (Figure 10c) enables detailed visualization of ridge–groove patterns and surface features, while the grayscale-enhanced image (Figure 10d) improves groove–ridge contrast for forensic analysis. To ensure comprehensive evaluation without overlooking major features, the LLP was divided into four quadrants: upper left, upper right, lower left, and lower right.^87^

**Figure 10:**
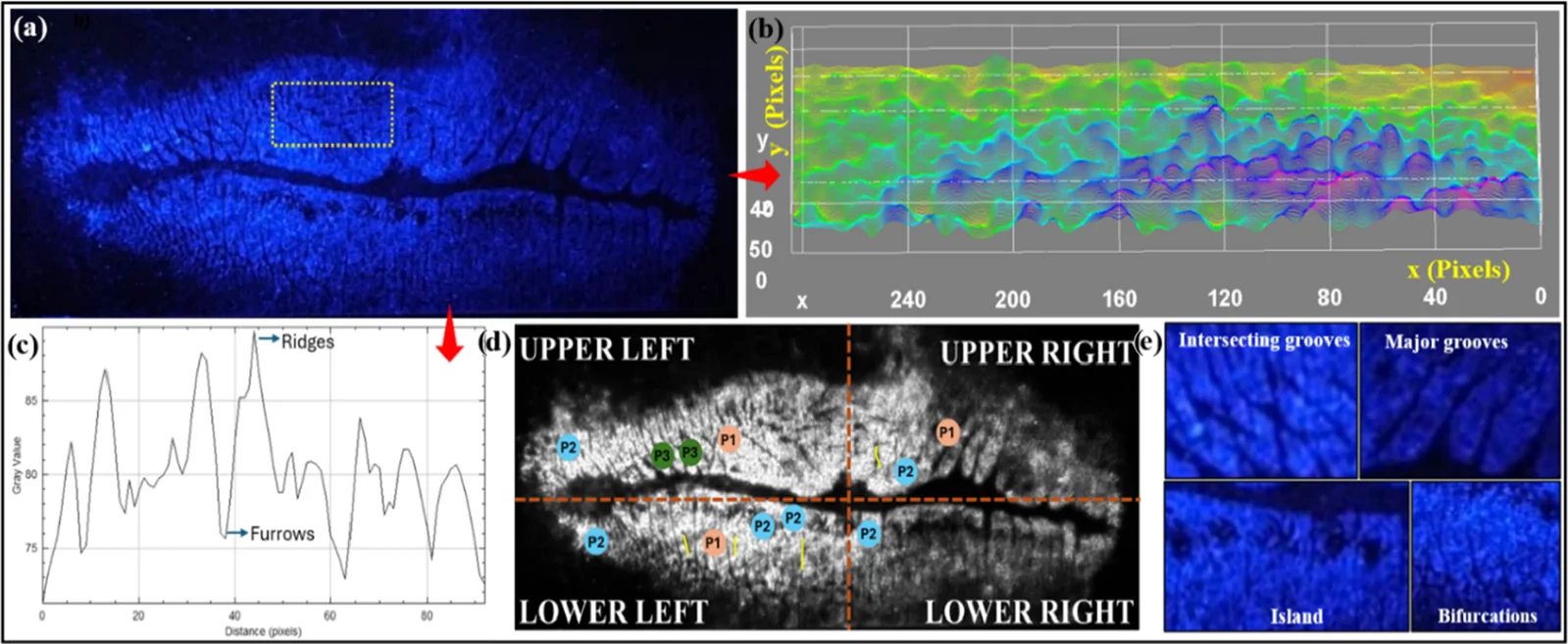
(a) Photograph of LLP under UV light developed using PNH on nonporous glass, (b) 3D interactive plot, and (c) grayscale pixel profile of lip print. (d) Grayscale-enhanced lip-print image and (e) magnified view of the characteristic groove patterns of the developed lip print for detailed analysis.

Minutiae analysis in Figure 10d identified bifurcation (P1), island (P2), and intersected/interacted groove (P3), demonstrating high-quality development and retention of detailed micromorphological features relevant to individual identification. Their accurate and reproducible visualization highlights the effectiveness of PNH in resolving distinctive lip-print patterns. The detailed groove architecture enables high-resolution mapping, while the well-defined vermilion border and 9.7 mm lip thickness, classified as medium according to Martin Santos (1967), further support its forensic utility. Figure 10e provides an enlarged view showing interacting and major grooves, islands, and bifurcations. Collectively, these qualitative and quantitative features demonstrate the reliability, repeatability, and evidentiary potential of PNH for precise lip-print identification, authentication, and forensic documentation.

### 3.10 Development of Aged Latent Prints

The ability of PNH to visualize aged LFPs and LLPs was evaluated under ambient conditions and environmental stresses (Figure S25). Prints stored for 1 day showed intense fluorescence with well-defined fingerprint ridges and lip-print grooves, indicating efficient interaction with freshly deposited residues. After 20 days, the fluorescence intensity slightly decreased, but ridge and groove patterns remained clearly distinguishable. This reduction is attributed to the evaporation and oxidative degradation of volatile and lipid components during aging, which reduces the residue available for interaction with PNH; nevertheless, sufficient sebaceous material remained to retain the powder and enable successful visualization.

The effects of environmental stress were further assessed by exposing undeveloped LFPs and LLPs to 70 °C and 92% RH before development. At 70 °C, moderate reduction in fluorescence intensity and image contrast were observed, likely due to thermal redistribution and partial lipid degradation, while 92% RH caused a slight reduction in contrast, probably from moisture-induced residue spreading. Despite these stresses, characteristic ridge and groove patterns remained clearly visible under UV illumination, demonstrating effective PNH visualization under adverse conditions. Overall, PNH imaging performance is governed primarily by preservation of the latent residue rather than degradation of the fluorescent material, supporting its suitability for practical forensic applications involving aged and environmentally exposed prints.

### 3.11 Comparative Evaluation of PNH with Recently Reported Multifunctional Fluorescent Materials for Anticounterfeiting and Forensic Applications

To assess the practical significance of PNH, its photophysical and forensic performance was benchmarked against recently reported multifunctional fluorescent materials for anticounterfeiting and LFP visualization (Table S3). PNH exhibits a distinctive combination of reversible optical responses within a single metal-free Schiff-base fluorophore. The fluorophore undergoes a pronounced mechanofluorochromic shift from 430/450 to 440/560 nm, corresponding to a blue-to-orange fluorescence transition that is recovered with DCM vapor. Notably, PNH exhibits a quantitative pressure sensitivity of 0.018–0.088 MPa, whereas pressure sensitivity was not reported for most of the compared materials. TPA-NC showed a 30 nm red shift, while Zn Schiff-base complexes exhibited a 457–545 nm shift with acetonitrile-assisted recovery. PNH also displays reversible acidofluorochromism, with emissions shifting from 425/449 to 453/532 nm upon HCl treatment and recovery with NH_3_. The switching stability was demonstrated over 50 HCl/NH_3_ cycles and 50 mechano-DCM cycles. In addition, the fluorescence intensity remained essentially unchanged after 12 h of continuous UV irradiation, demonstrating good photostability. Although several reported materials show good photostability or light fastness, quantitative irradiation-based data were not consistently available.

For forensic applications, PNH extends beyond conventional fluorescent powders by providing high-contrast visualization of LFPs and LLPs across diverse and challenging substrates, including glass, glossy paper, CDs, and white ceramic tiles with a matching background. It enables visualization of level 1–3 fingerprint characteristics, including ridge patterns, endings, bifurcations, and sweat pores, supported by grayscale intensity and 3D surface analyses. This compares favorably with reported materials such as FNH, BTNH, BTAF, CNS, and OAF, which demonstrate variable level 1–3 visualization depending on the substrate. PNH also successfully develops lip-print groove architecture and minutiae such as bifurcations, islands, and intersected grooves. Importantly, molecular dynamics and FTIR studies provide molecular-level evidence of interactions with glycine and myristic acid, representative fingerprint constituents, on hydrophilic and hydrophobic SiO_2_ surfaces—an aspect rarely addressed for related fluorescent powders. Its ability to retain performance on aged and environmentally stressed prints, together with strong ridge–furrow contrast and performance comparable or superior to commercial fingerprint powder, further enhances its practical value. Overall, PNH combines reversible mechanofluorochromism and acidofluorochromism, quantified pressure sensitivity, excellent switching durability, prolonged photostability, and level 1–3 fingerprint visualization within a single metal-free fluorophore. Together with its eco-friendly water-based ink formulation, these features highlight its potential for advanced security printing and forensic authentication.

## 4 Conclusion

A structurally hybrid fluorophore, PNH, incorporating phenanthrene and naphthol frameworks, was synthesized through a simple one-step route and was structurally confirmed by spectroscopic techniques. PNH exhibits a combination of aggregation-induced emission, intramolecular charge transfer, reversible mechano- and acidofluorochromism, and good photostability, highlighting its potential as a multifunctional pigment for security inks and latent finger and lip-print visualization. Quantitative pressure-dependent studies established its mechanofluorochromic response, while 50-cycle switching experiments demonstrated excellent reversibility and cycling stability. Water-based PNH screen prints showed strong UV resistance and mechanical durability while incorporating multiple orthogonal security features, including daylight invisibility, mechanofluorochromism, acid/base-vapor-responsive fluorescence, and THF-induced rear-side fluorescence, supporting their potential for reusable anticounterfeiting applications.

FTIR and molecular-interaction studies revealed robust supramolecular hydrogen-bonding interactions between PNH and both hydrophilic and hydrophobic fingerprint constituents, facilitating selective adherence to fingerprint ridges across nonporous, semiporous, challenging, and same-background substrates. Consequently, PNH produced high-contrast LFPs with clearly resolved level 1–3 features, further validated by grayscale pixel intensity profiling and 3D interactive surface mapping. PNH also enabled clear visualization of characteristic lip-print grooves and minutiae, demonstrating its potential for cheiloscopic applications. Its effectiveness was retained for aged latent impressions and prints exposed to an elevated temperature and high humidity, despite some changes in fluorescence contrast. Overall, the facile one-step synthesis, multifunctional photophysical behavior, robust security features, broad substrate compatibility, and effective visualization of both latent fingerprints and lip prints establish PNH as a versatile, metal-free multifunctional platform for advanced security printing, reusable anticounterfeiting, and forensic latent-print visualization.

## Supplementary Material









## Data Availability

All data generated or analyzed during this study are included in this published article and its Supporting Informationfiles.

## Notes

Supporting Figures: FTIR, ^1^H NMR, ^13^C NMR, and mass spectrum of PNH; emission spectra of PNH exposed to 390 nm UV radiation for different durations; dose–response curve of PNH in HEK293T cells using MTT assay; atomic structure of optimized structures of single, dimer, and trimer PNH in the gas phase and solution phase; ESP surface of PNH along with the atomic notations from the gas-phase and solution-phase geometry optimization; chromaticity plot of PNH in THF–hexane mixtures; particle size distribution of PNH in 0% and 90% water fractions in THF–water mixture; FTIR of PNH and HCl-fumed PNH; SEM images of PNH and PNH-g; photographs and normalized PL spectra of PNH pellets prepared under different applied pressures; CIELAB color space with colorimetric values for blank and print, prints before and after UV exposure, and prints before and after rub test; THF-treated printed papers under 390 nm UV light; chromaticity plot of print, and HCl-fumed and NH_3_-fumed prints; fluorescence intensity retention and PL spectra of HCl-fumed and NH3-recovered printed patterns during 50 consecutive HCl/NH3 vapor switching cycles; fluorescence intensity retention and PL spectra of the rubbed and DCM-vapor-recovered prints during 50 consecutive mechanofluorochromic switching cycles; FTIR spectral comparison; comparison of LFP visualization using a commercial fingerprint powder and PNH; 3D surface profilometry image of a LFP developed on a CD surface; and development of aged LFPs and LLPs using PNH under different storage and environmental conditions; Supporting tables: Atomic charges of the PNH molecule in the gas and solution-phase geometry optimization; and comparison of PNH with recently reported multifunctional fluorescent materials for anticounterfeiting and forensic applications (PDF)
Mechanofluorochromism in print (MP4)
H-terminated SiO_2_ surface (MP4)
Methyl-terminated SiO_2_ surface (MP4)
