# Supplementary material for: Stimuli-Responsive Phenanthrene–Naphthol Fluorescent Marker for Security and Forensic Authentication
Source: ACS Omega. 2026 Oct 21;11(43):66724–42. doi: 10.1021/acsomega.6c06304 (PMC13736858; doi:10.1021/acsomega.6c06304)
Supplement: Supplementary file 1 [file ao6c06304_si_001.pdf]

Stimuli Responsive Phenanthrene-Naphthol Fluorescent Marker for Security and Forensic Authentication

Rakshitha K Jain[a], Anand P J[a,b], Dhanya Sunil[a*], Poornima Bhagavath[a], Anoop Kishore Vatti[a], Shamanth Yagati Udaykumar[a]

[a] Manipal Institute of Technology, Manipal Academy of Higher Education, Manipal, India
[b] Future Products Industrial Company, Dammam 31411, Kingdom of Saudi Arabia

No.	Content	Page No.
**SF 1**	FTIR spectrum of PNH	3
**SF 2**	^{1}H NMR spectrum of PNH in DMSO-d_6	4
**SF 3**	^{13}C NMR spectrum of PNH in DMSO-d_6	4
**SF 4**	Mass spectrum of PNH	5
**SF 5**	PL spectra of PNH exposed to 390 nm UV radiation for different durations; λ_{ex} = 390 nm	5
**SF 6**	Dose-response curve of PNH in HEK293T cells using MTT assay	6
**SF 7**	Atomic structure of optimized structures a) single, b) dimer and c) trimer PNH in the gas phase	6
**SF 8**	Atomic structure of optimized structures a) single, b) dimer and c) trimer PNH in the solution phase.	6
**SF 9**	The ESP surface of PNH along with the atomic notations from the gas-phase geometry optimization.	7
**ST 1**	The atomic charges of the PNH molecule in the gas-phase geometry optimization	7
**SF 10**	The ESP surface of PNH along with the atomic notations from solution-phase geometry optimization.	8
**ST 2**	The atomic charges of the PNH molecule in the solution-phase geometry optimization	8
**SF 11**	Chromaticity plot of PNH in THF-hexane mixtures (1×10^{-4}); λ_{ex} =390 nm.	9

**SF 12**	Particle size distribution of PNH in a) 0% and b) 90% water fraction in THF-water mixture.	9
**SF 13**	FTIR of PNH and HCl fumed PNH	10
**SF 14**	SEM images of a) PNH and b) PNH-g	10
**SF 15**	Photographs of PNH pellets prepared under different applied pressures (0.018-0.088 MPa).	10
**SF 16**	Normalized PL spectra of PNH pellets prepared under controlled compressive pressures (0.018-0.088 MPa).	11
**SF 17**	CIELAB color space with colorimetric values for a) blank and print, b) prints before and after UV exposure (300 W/30 m/min) and c) prints before and after rub test.	11
**SF 18**	THF treated printed papers under 390 nm UV light: a) front and b) rear sides.	11
**SF 19**	Chromaticity plot of print, and HCl fumed & NH_3 fumed prints; λ_{ex} = 390 nm.	12
**SF 20**	a) Fluorescence intensity retention of the HCl-fumed and NH_3-recovered printed patterns during 50 consecutive HCl/NH_3 vapor switching cycles and b) PL spectra of the HCl-fumed and NH_3-recovered printed patterns recorded after 10, 20, 30, 40, and 50 switching cycles.	12
**SF 21**	a) Fluorescence intensity retention of the rubbed and DCM-vapor-recovered prints during 50 consecutive mechanofluorochromic switching cycles and b) PL spectra of the rubbed and DCM-vapor-recovered printed patterns recorded after 10, 20, 30, 40, and 50 switching cycles.	12
**SF 22**	FTIR spectral comparison of a) glycine, water, glycine + water, PNH, and glycine + water + PNH, b) enlarged view of the highlighted spectral region in (a), c) myristic acid, water, myristic acid + water, PNH, and myristic acid + water + PNH, (d) enlarged view of the highlighted spectral region in (c), e) glycine, water, glycine + water, myristic acid + water, PNH, and glycine + myristic acid + water + PNH; and (f) enlarged view of the highlighted spectral region in (e). G- Glycine, MA- Myristic acid, W- Water.	13

**SF 23**	Comparison of LFP visualization using a commercial fingerprint powder and PNH under UV illumination, along with the corresponding grayscale intensity profiles.	14
**SF 24**	3D surface profilometry image of a LFP developed on a CD surface.	14
**SF 25**	Development of aged LFPs and LLPs using PNH under different storage and environmental conditions.	15
**ST 3**	Comparison of PNH with recently reported multifunctional fluorescent materials for anti-counterfeiting and forensic applications.	15

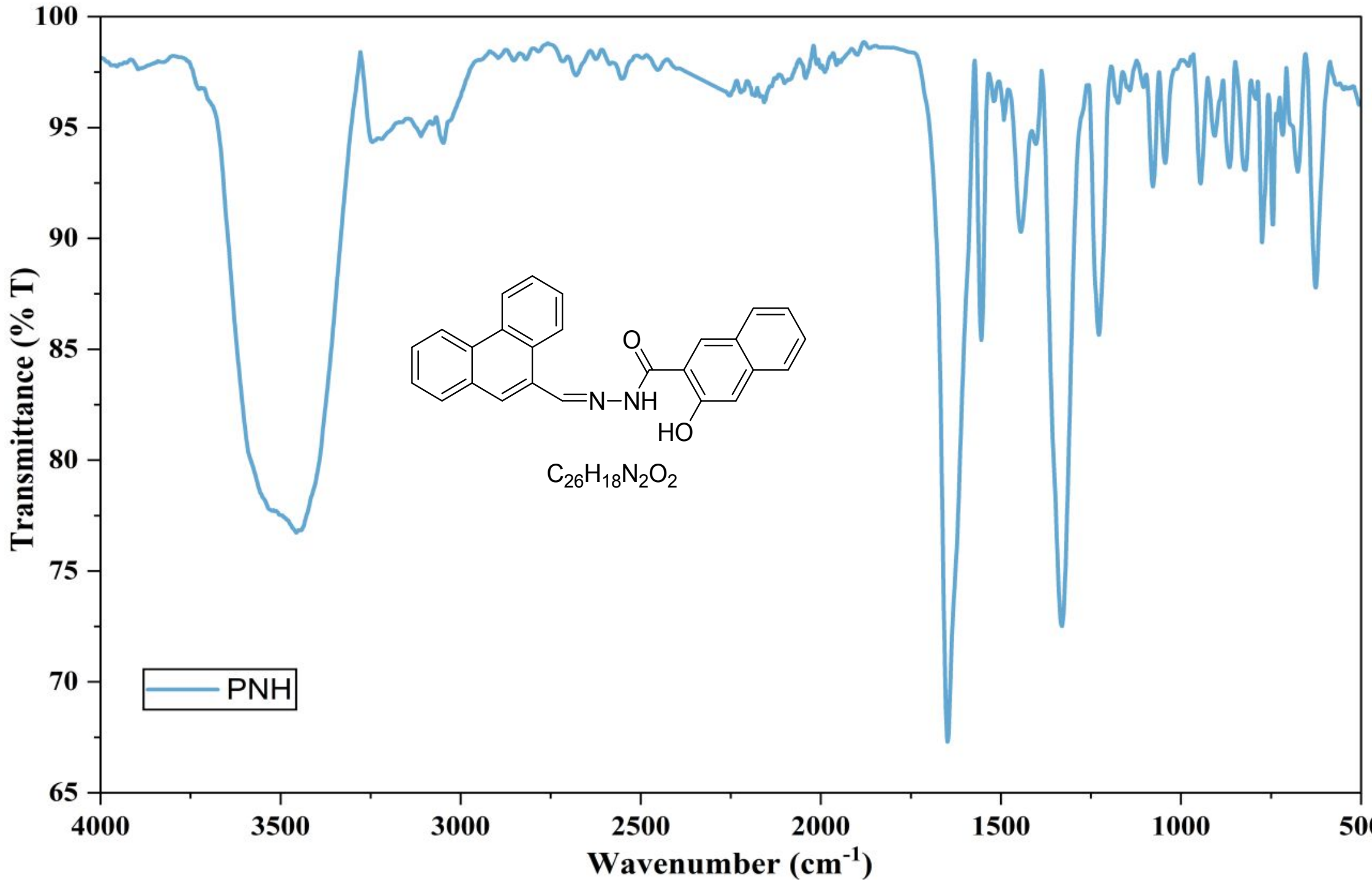

SF 1: FTIR spectrum of PNH.

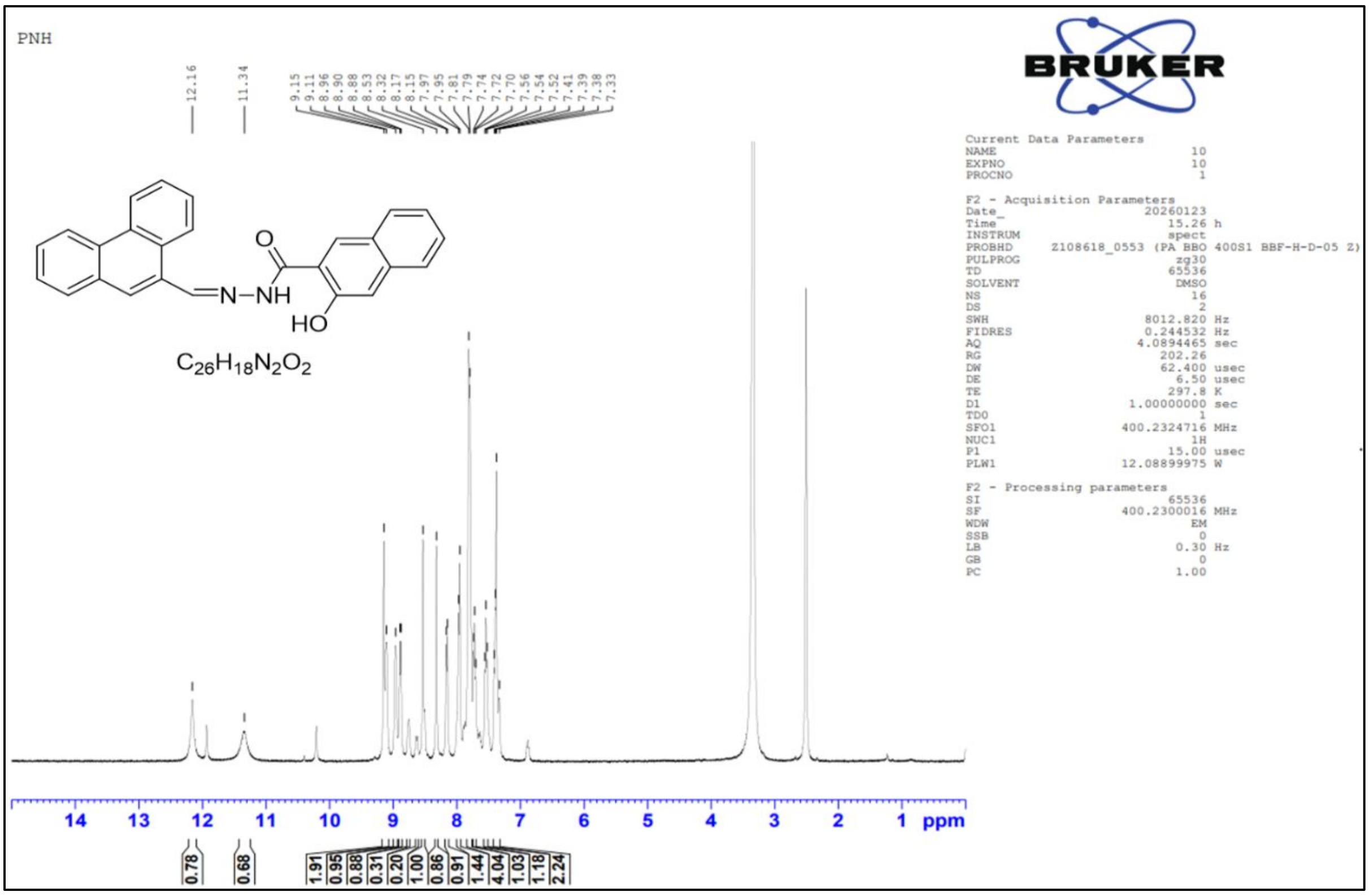

SF 2: ^{1}H NMR spectrum of PNH in DMSO-d_6.

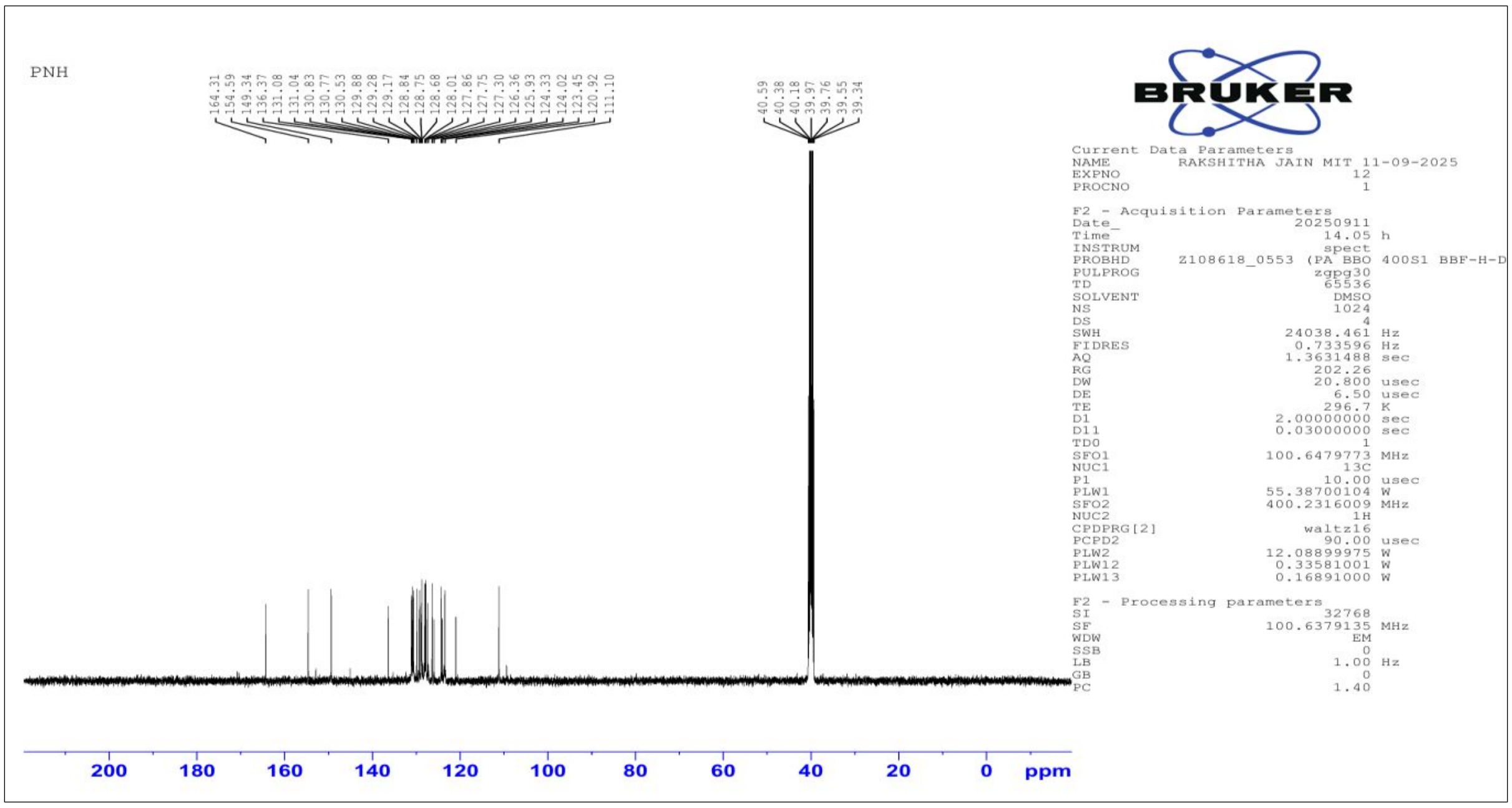

SF 3: ^{13}C NMR spectrum of PNH in DMSO-d_6.

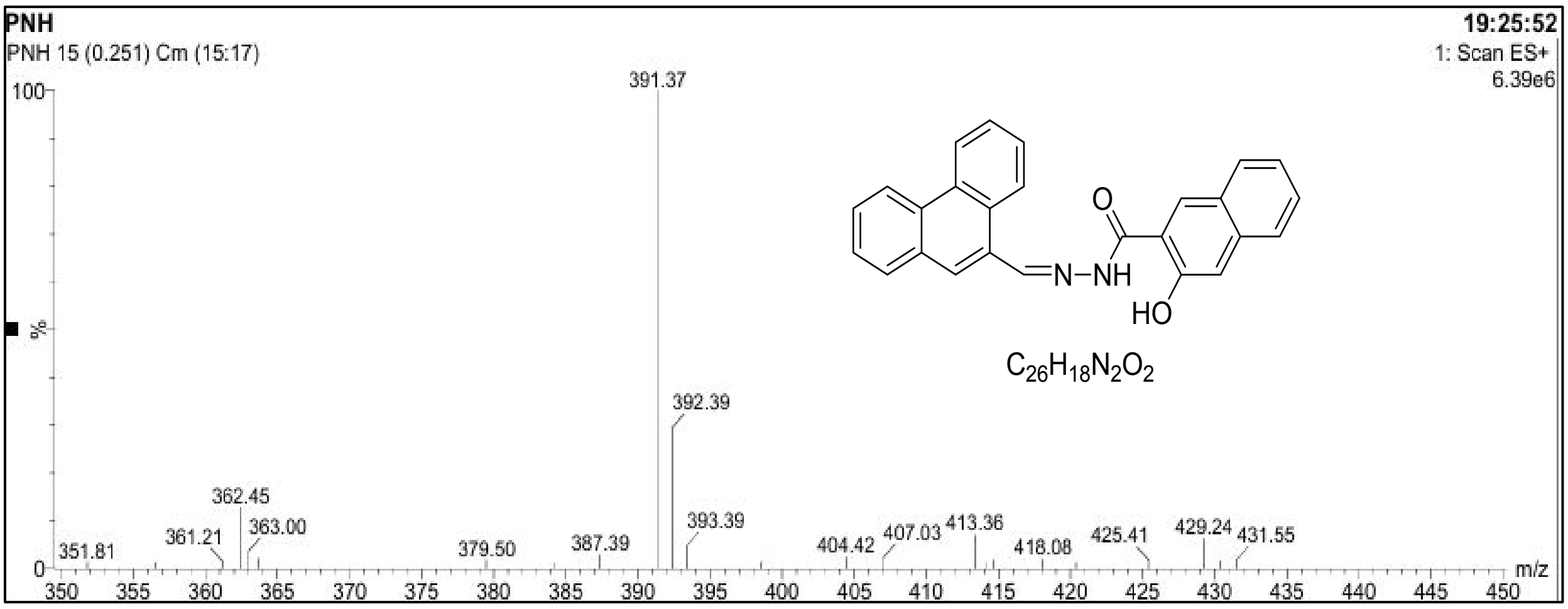

SF 4: Mass spectrum of PNH.

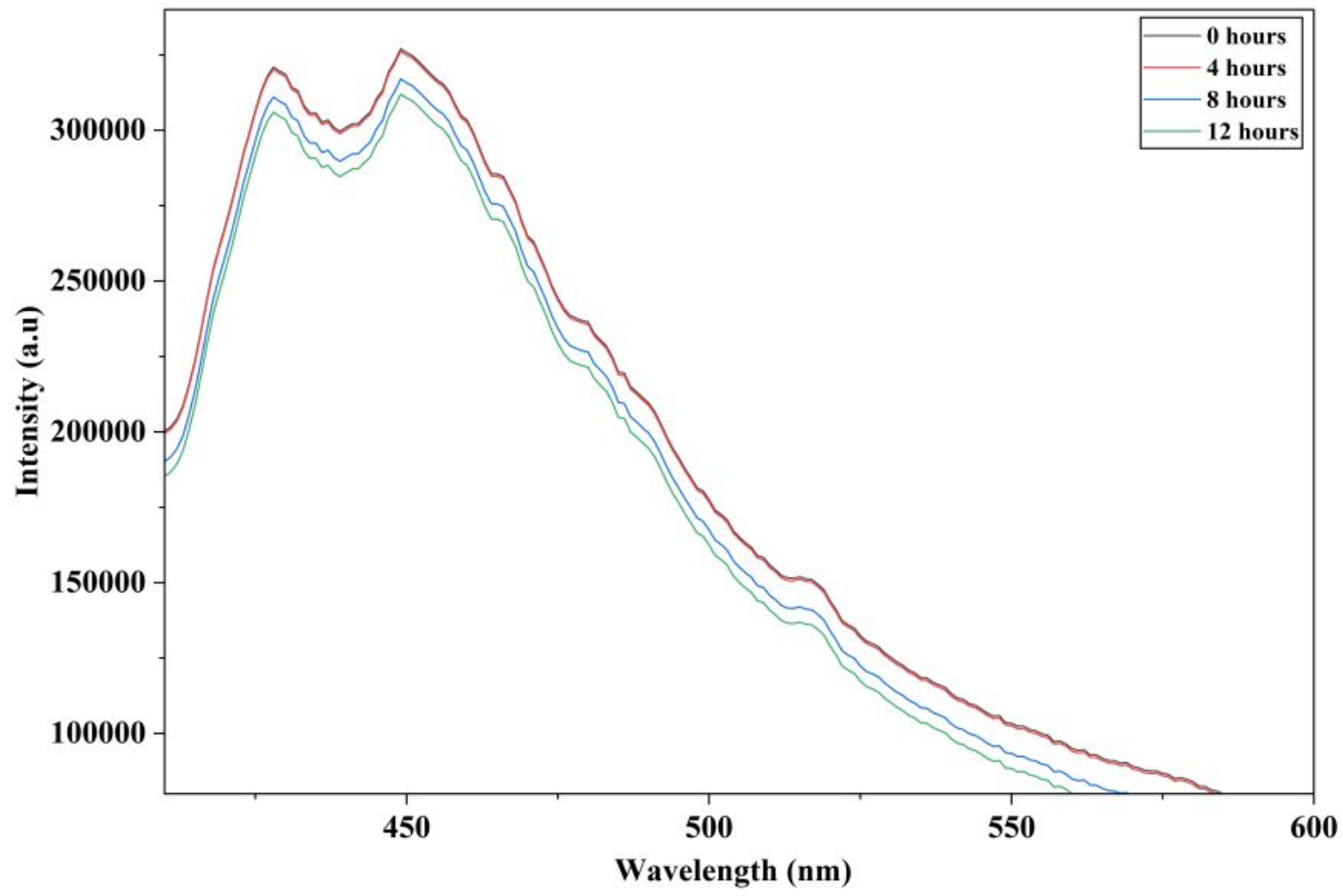

SF 5: PL spectra of PNH exposed to 365 nm UV radiation for different durations; λ_{ex} = 390 nm.

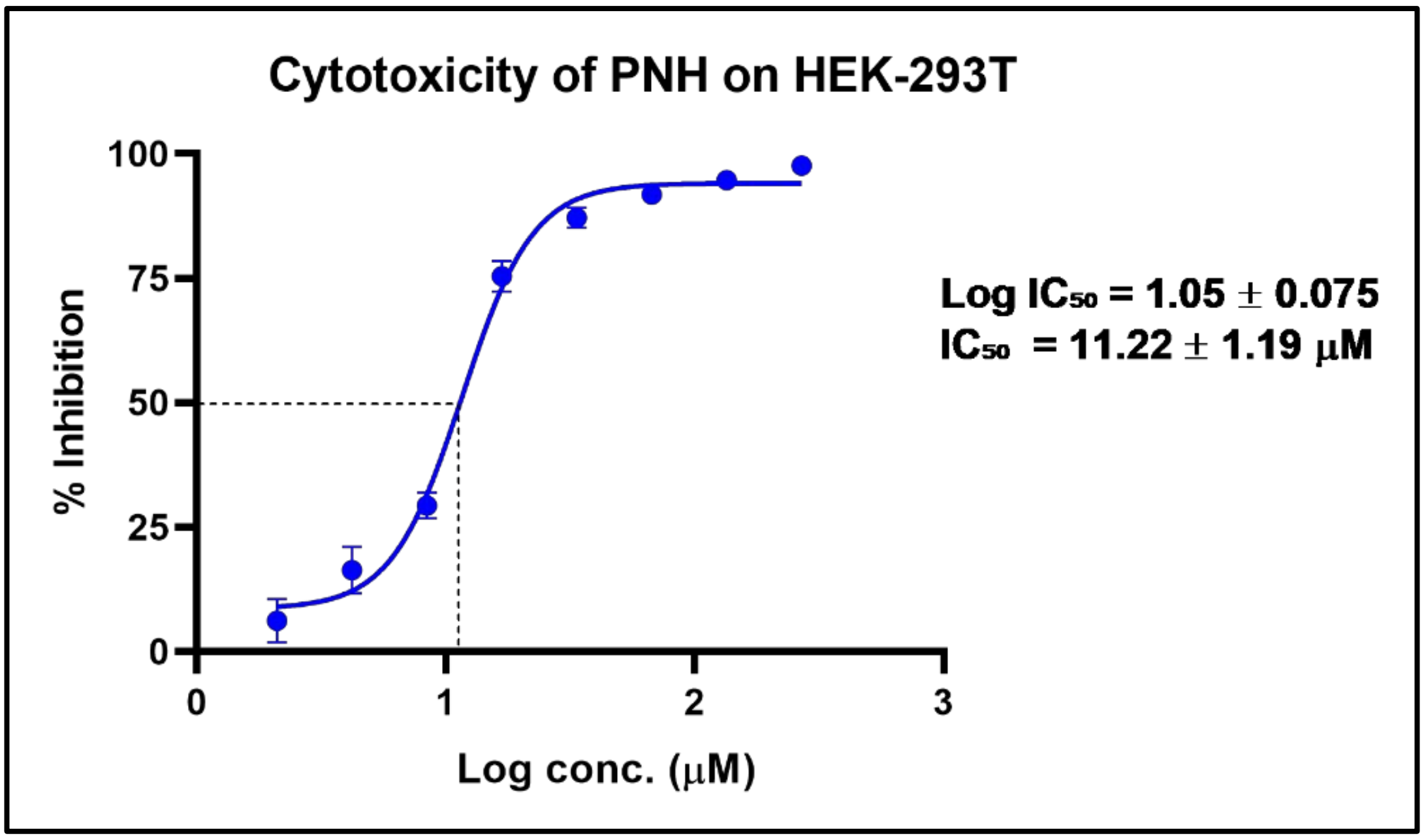

SF 6: Dose-response curve of PNH in HEK293T cells using MTT assay.

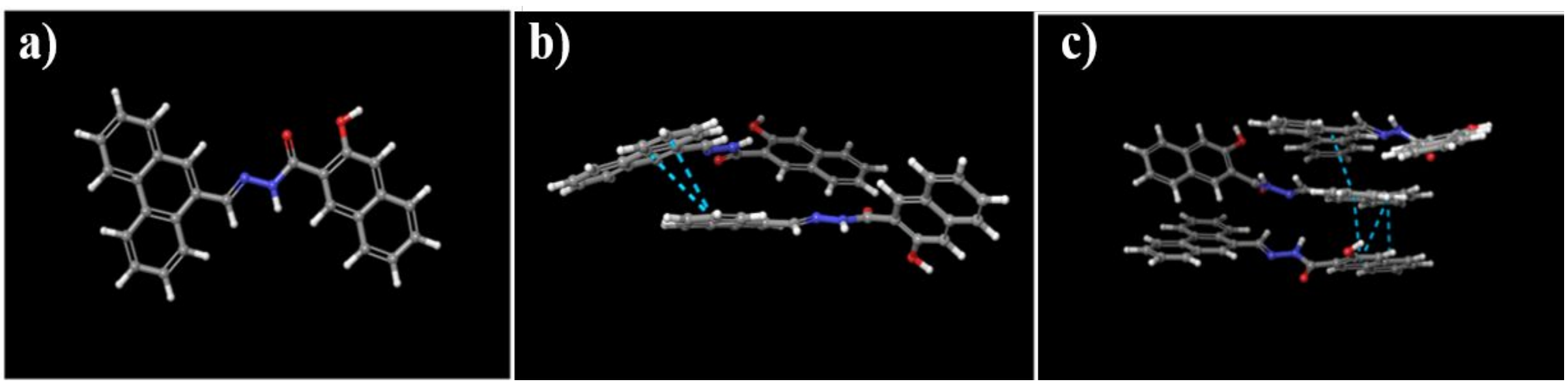

SF 7: Atomic structure of optimized structures a) single, b) dimer and c) trimer PNH in the gas phase. π-π bonding is shown with blue dashed line, oxygen in red, nitrogen in blue, carbon in grey, and hydrogen in white.

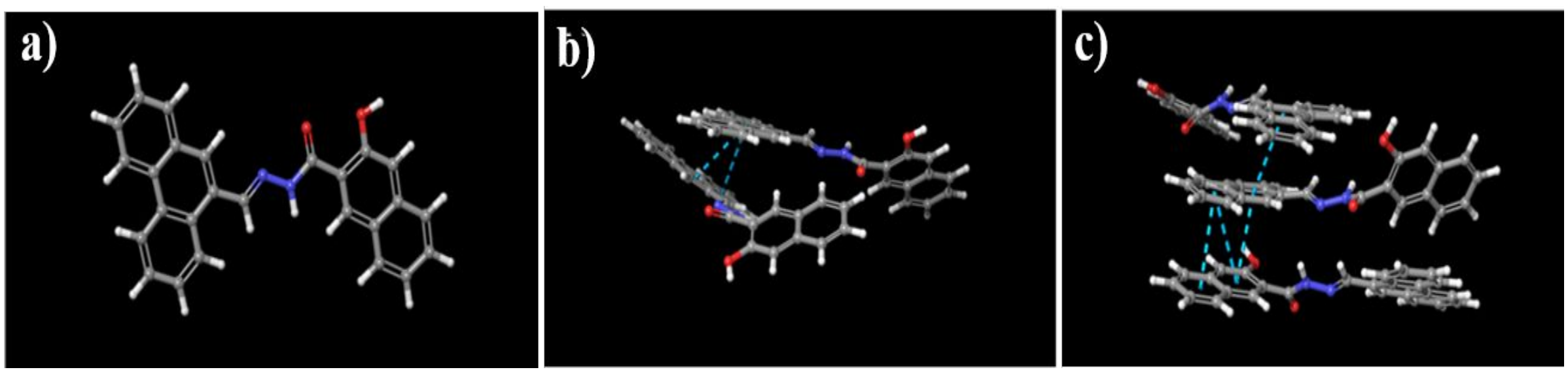

SF 8: Atomic structure of optimized structures a) single, b) dimer and c) trimer PNH in the solution phase. π-π bonding is shown with blue dashed line, oxygen in red, nitrogen in blue, carbon in grey, and hydrogen in white.

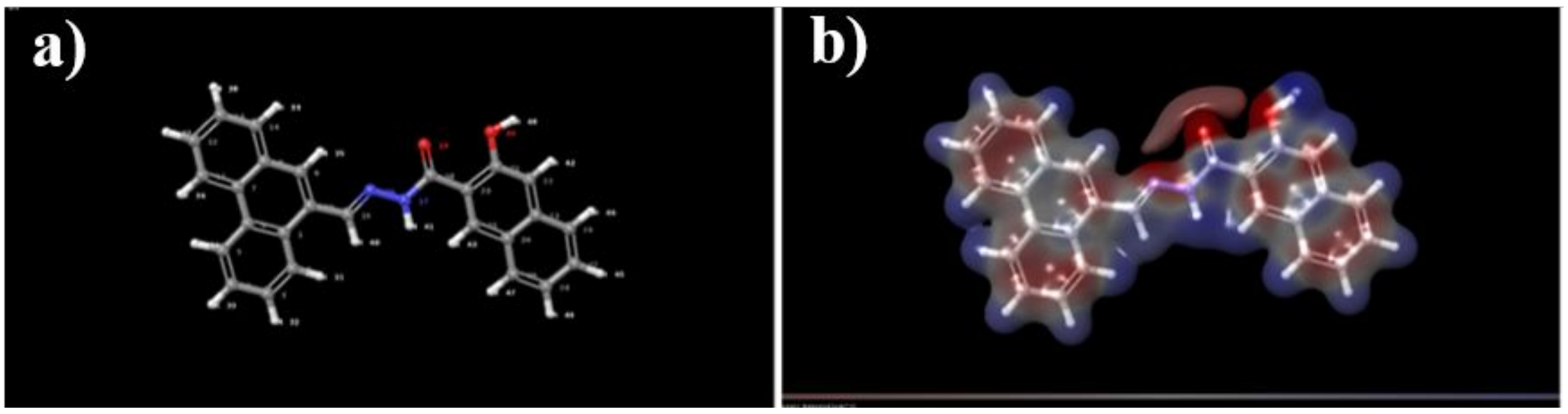

SF 9: The ESP surface of PNH along with the atomic notations from the gas-phase geometry optimization. The colour coding is as follows: oxygen is in red, nitrogen is in blue, carbon is in grey, and hydrogen is in white.

ST 1: The atomic charges of the PNH molecule in the gas-phase geometry optimization.

PNH atoms	Atomic Charges	PNH atoms	Atomic Charges	PNH atoms	Atomic Charges
C1	0.27796	N17	-0.31283	H33	0.12789
C2	-0.32844	C18	0.67295	H34	0.12665
C3	-0.04378	O19	-0.47619	H35	0.08526
C4	-0.17101	C20	-0.23136	H36	0.13349
C5	-0.12700	C21	0.50300	H37	0.11849
C6	-0.04208	C22	-0.59020	H38	0.11607
C7	0.03567	C23	0.29698	H39	0.16107
C8	0.15598	C24	0.21041	H40	0.03153
C9	-0.07068	C25	-0.34720	H41	0.25509
C10	-0.28726	C26	-0.26827	H42	0.19829
C11	-0.18141	C27	-0.11461	H43	0.17709
C12	-0.11890	C28	-0.10459	H44	0.15610
C13	-0.08947	C29	-0.28149	H45	0.12989
C14	-0.24792	O30	-0.52874	H46	0.12513
C15	0.25873	H31	0.16262	H47	0.15768
N16	-0.22622	H32	0.10928	H48	0.40639

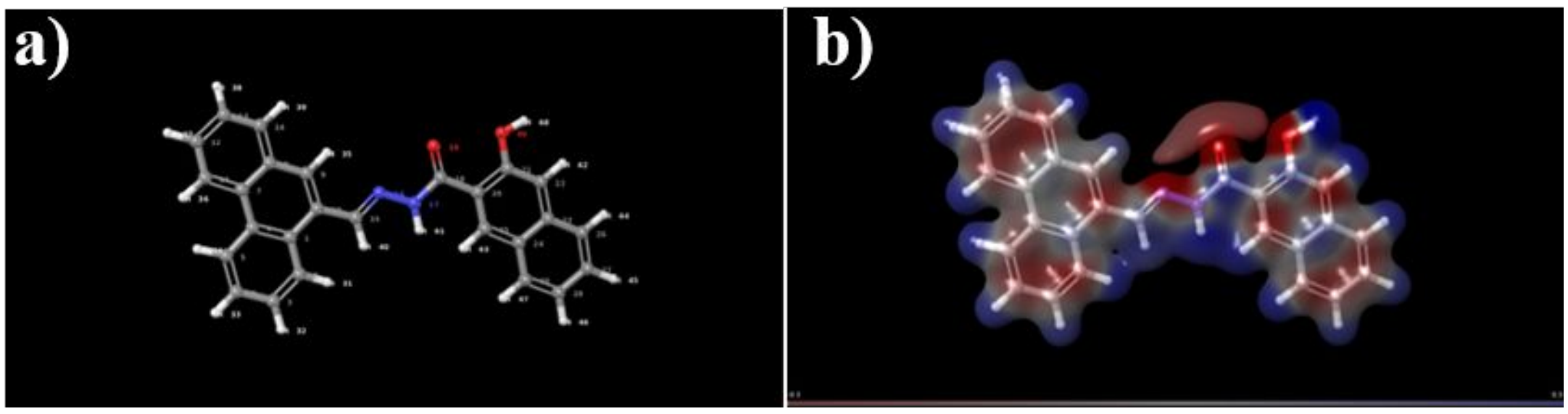

SF 10: The ESP surface of PNH along with the atomic notations from solution-phase geometry optimization. The colour coding is as follows: oxygen is in red, nitrogen is in blue, carbon is in grey, and hydrogen is in white.

ST 2: The atomic charges of the PNH molecule in the solution-phase geometry optimization.

PNH atoms	Atomic Charges	PNH atoms	Atomic Charges	PNH atoms	Atomic Charges
C1	0.30112	N17	-0.26616	H33	0.14579
C2	-0.36808	C18	0.67236	H34	0.13634
C3	-0.02269	O19	-0.55635	H35	0.08164
C4	-0.20218	C20	-0.24435	H36	0.15111
C5	-0.11521	C21	0.56077	H37	0.13154
C6	-0.07337	C22	-0.65965	H38	0.12725
C7	0.05598	C23	0.30871	H39	0.17365
C8	0.13785	C24	0.25028	H40	0.05286
C9	-0.07332	C25	-0.39411	H41	0.28232
C10	-0.33200	C26	-0.28984	H42	0.23872
C11	-0.20482	C27	-0.12971	H43	0.21388
C12	-0.12093	C28	-0.09502	H44	0.17480
C13	-0.09705	C29	-0.33063	H45	0.14340
C14	-0.25763	O30	-0.58439	H46	0.13323
C15	0.31972	H31	0.18702	H47	0.18613
N16	-0.30744	H32	0.11841	H48	0.44006

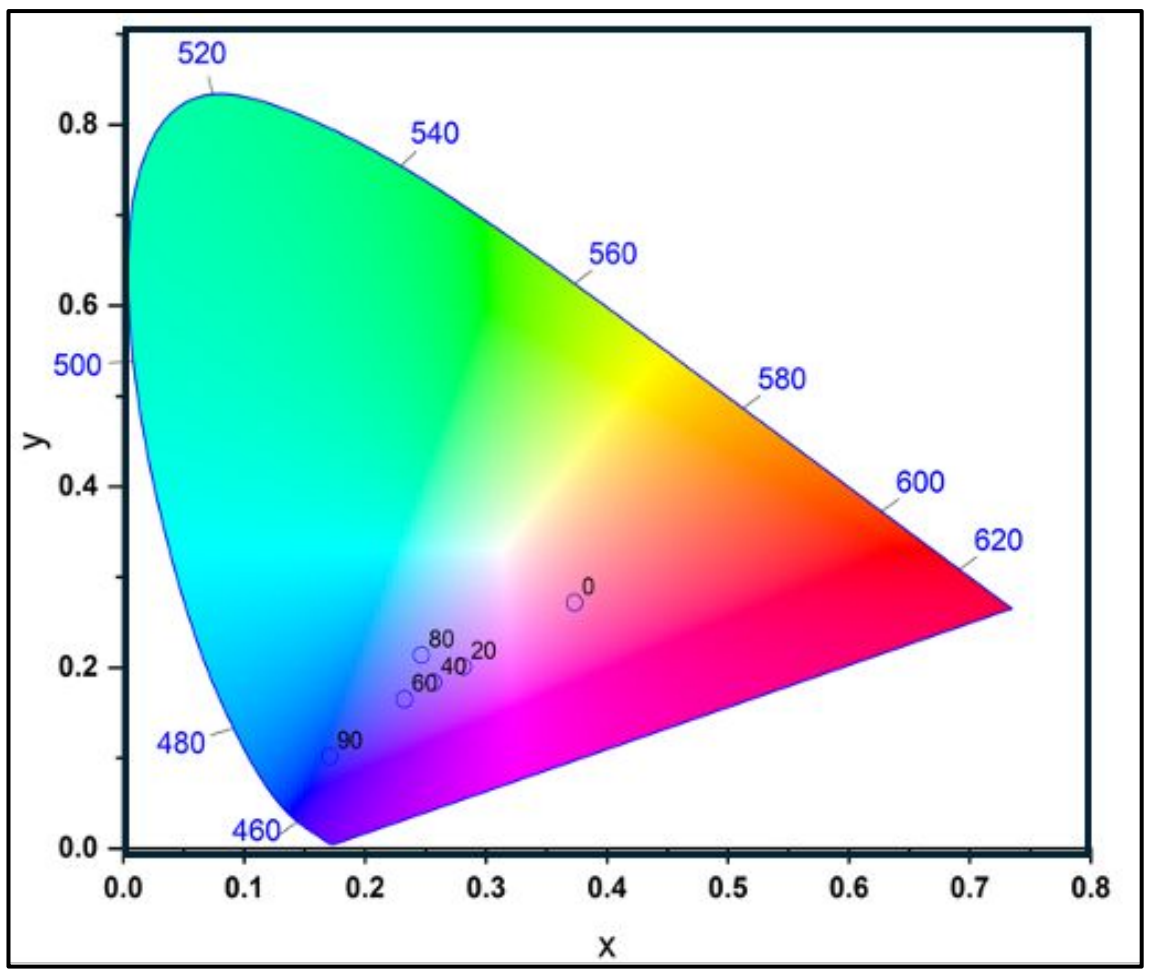

SF 11: Chromaticity plot of PNH in THF-hexane mixtures (1×10^{-4}); λ_{ex} =390 nm.

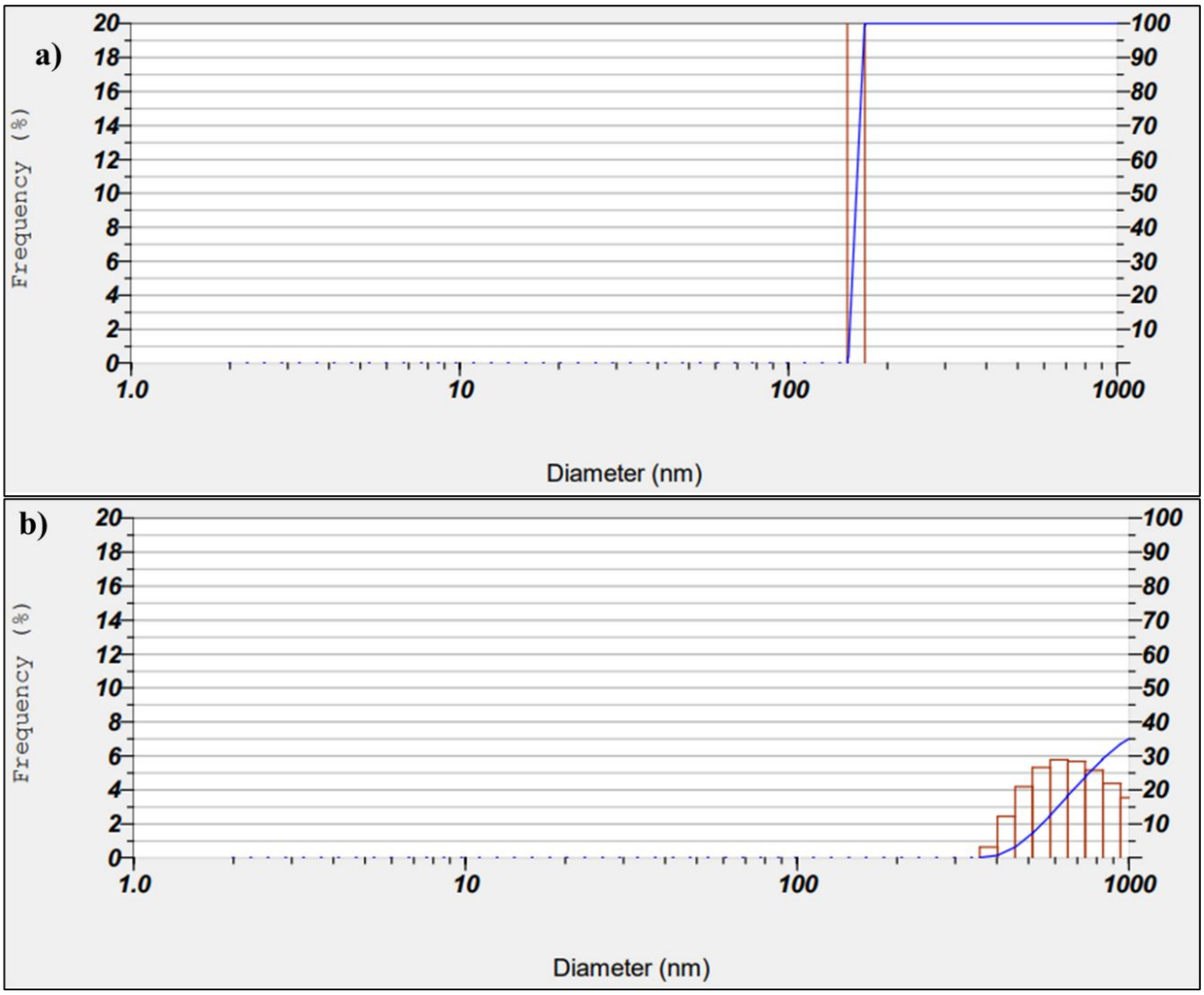

SF 12: Particle size distribution of PNH in a) 0% and b) 90% water fraction in THF-water mixture.

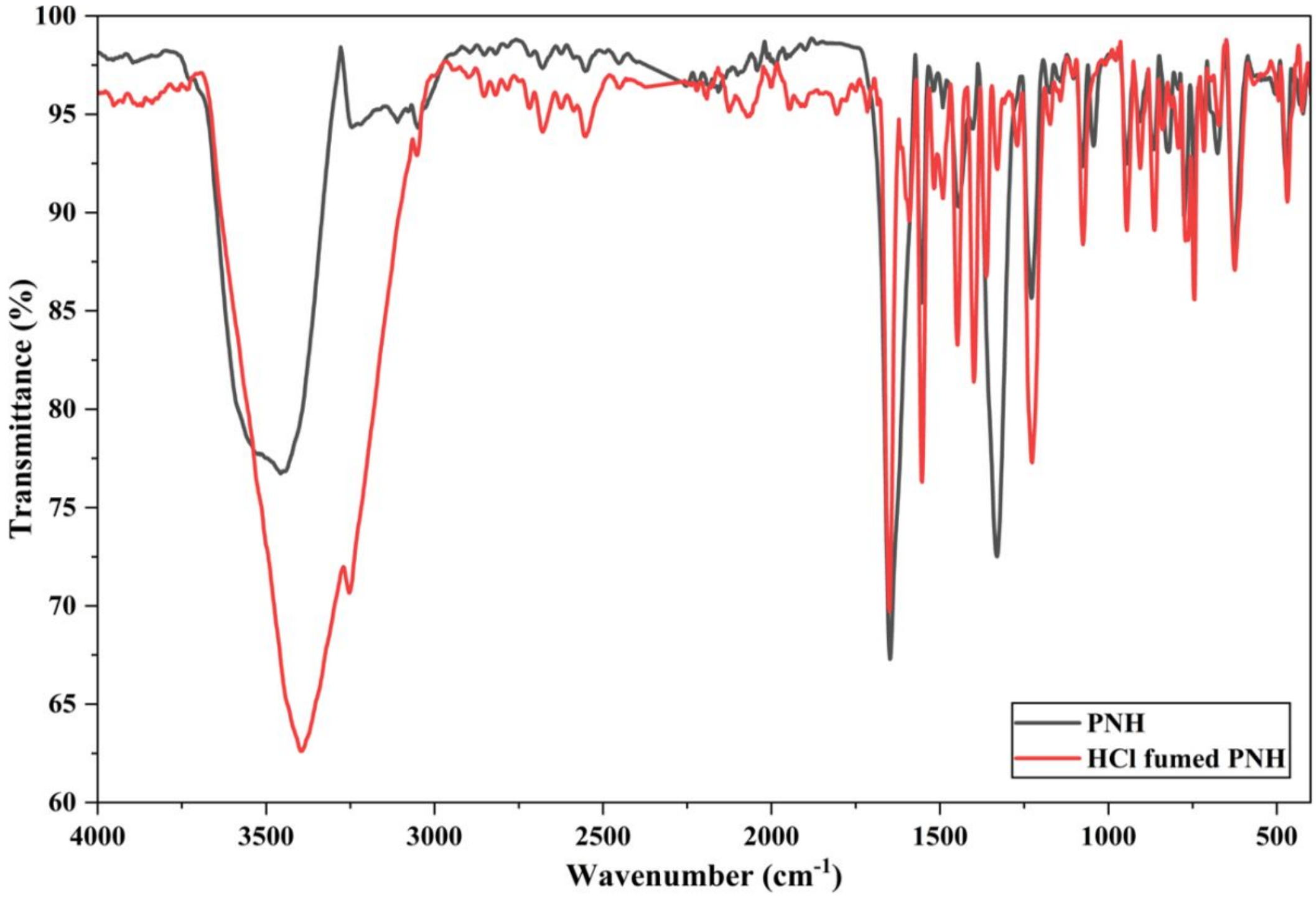

SF 13: FTIR of PNH and HCl fumed PNH.

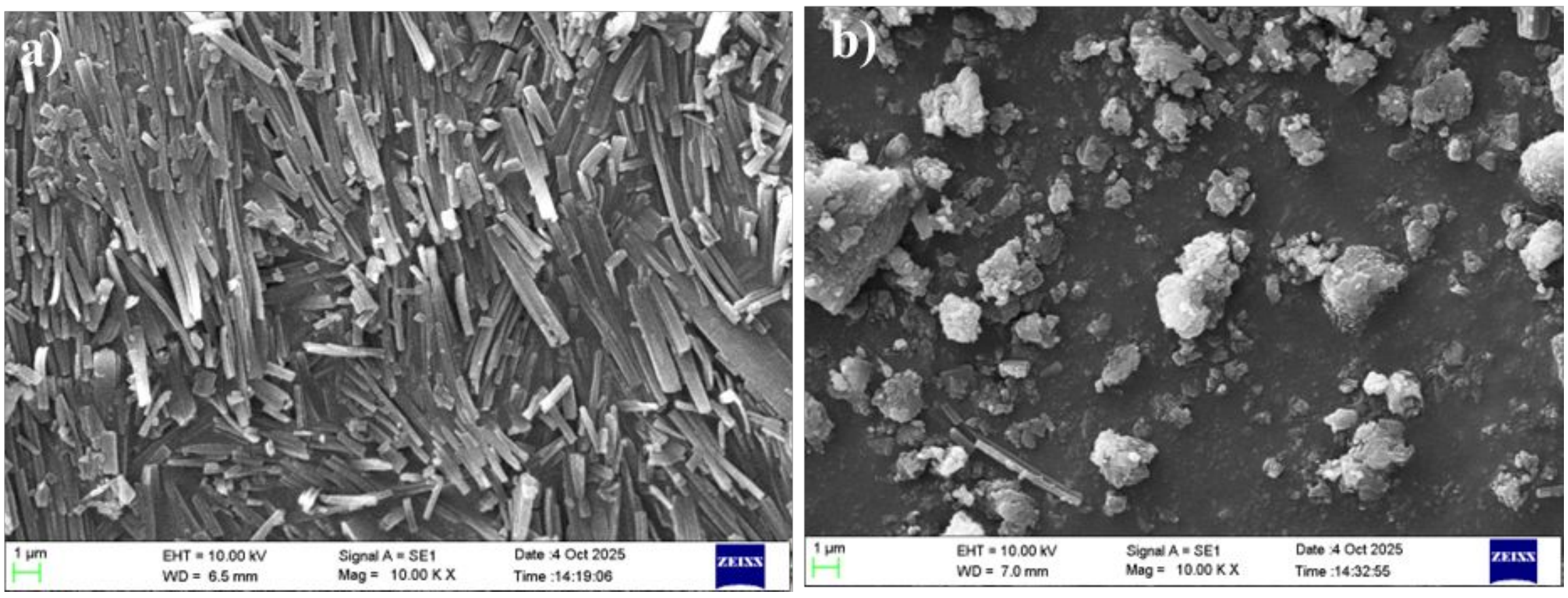

SF 14: SEM images of a) PNH and b) PNH-g.

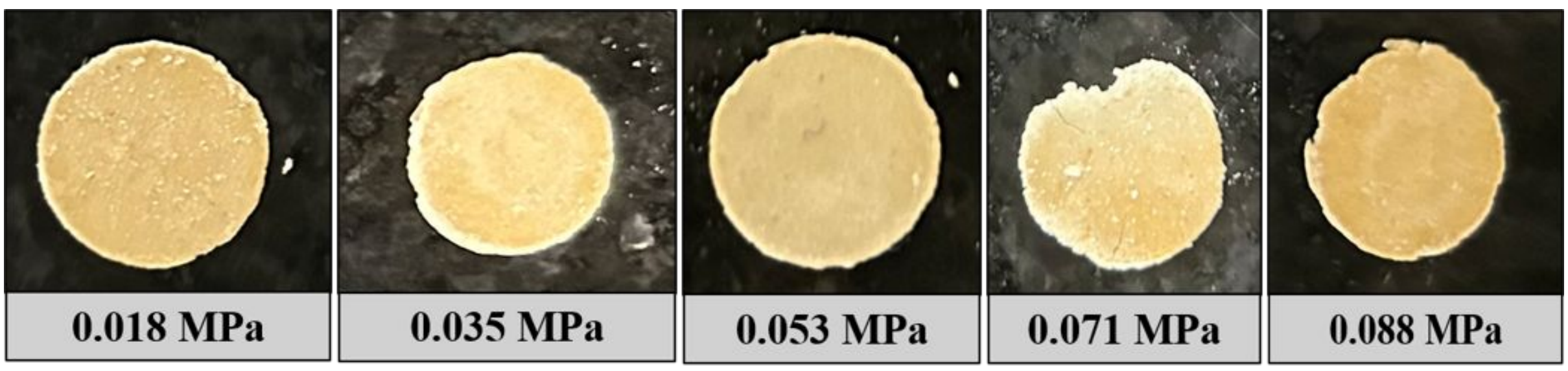

SF 15: Photographs of PNH pellets prepared under different applied pressures (0.018-0.088 MPa).

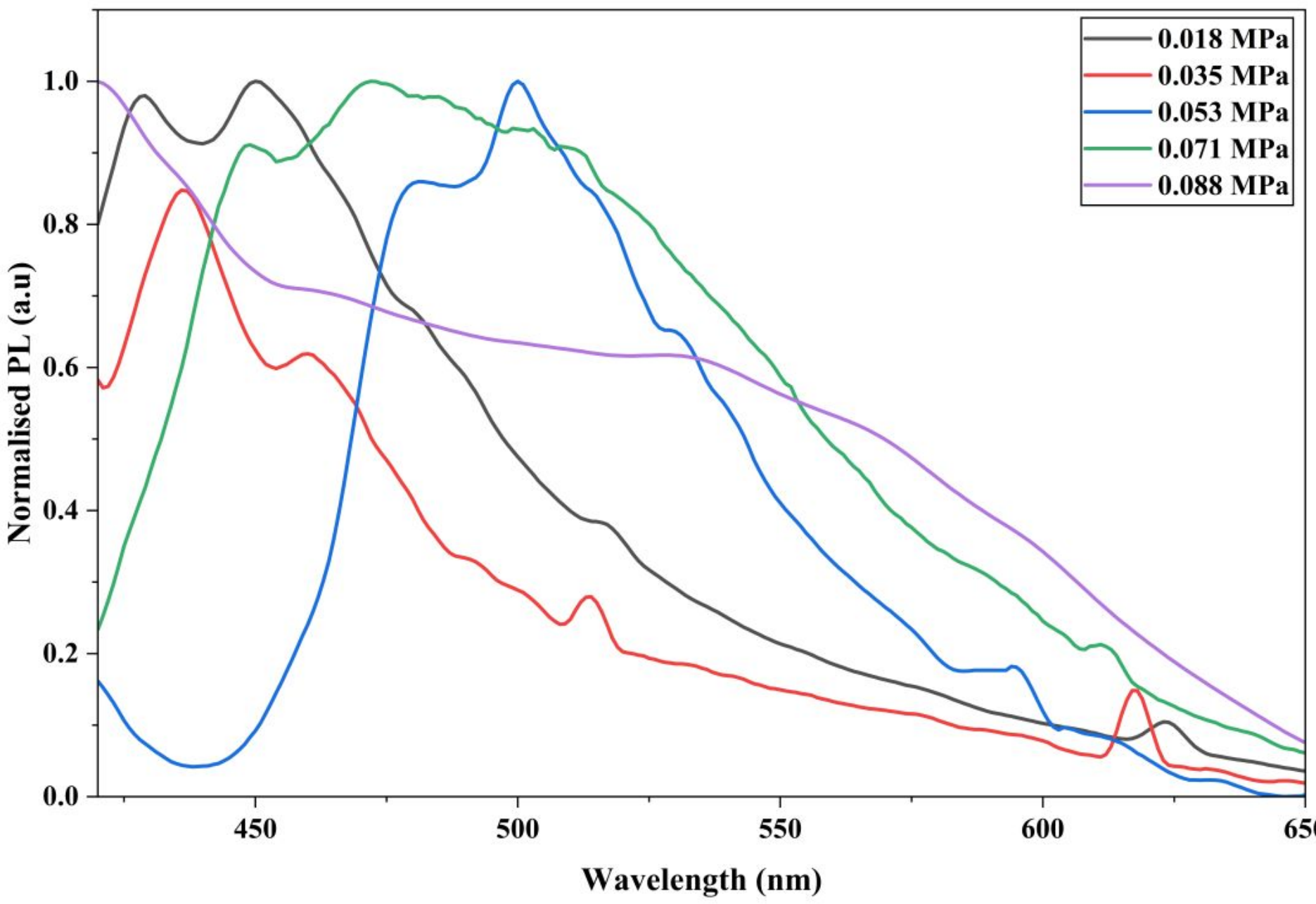

SF 16: Normalized PL spectra of PNH pellets prepared under controlled compressive pressures (0.018-0.088 MPa).

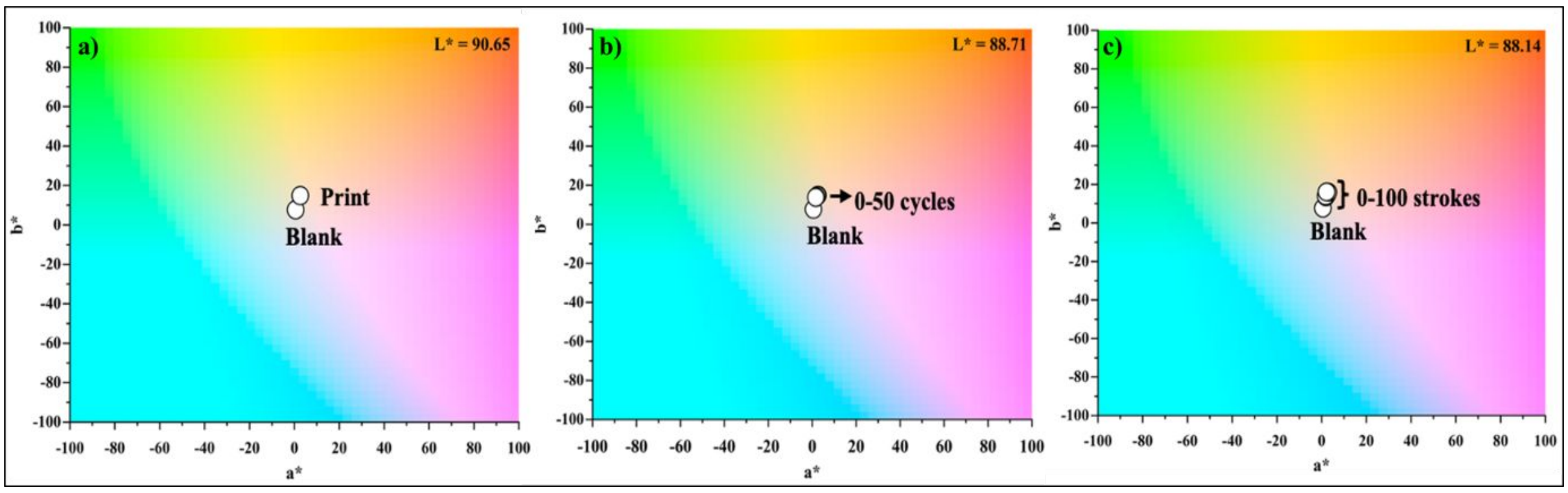

SF 17: CIELAB color space with colorimetric values for a) blank and print, b) prints before and after UV exposure (300 W/30 m/min) and c) prints before and after rub test.

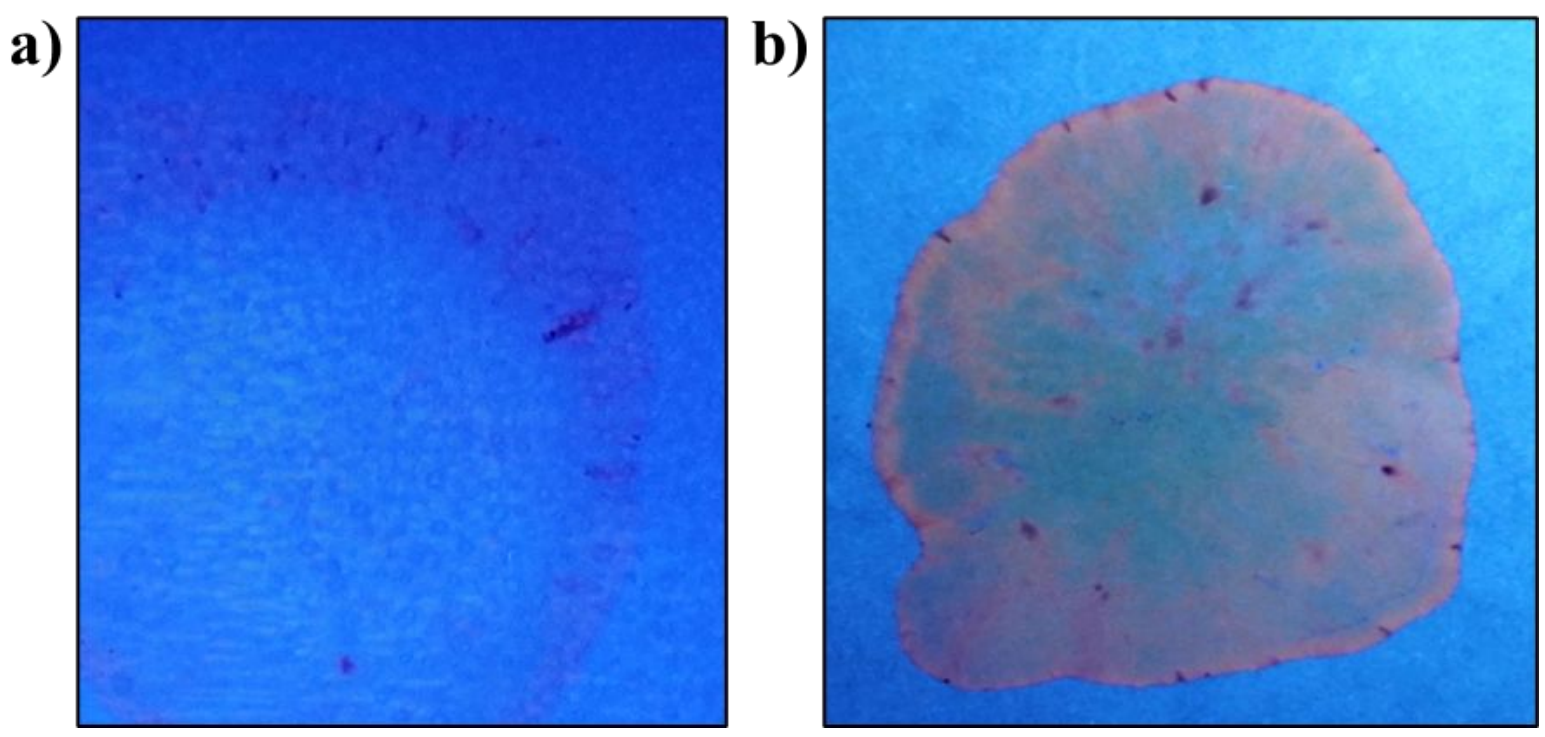

SF 18: THF treated printed papers under 390 nm UV light: a) front and b) rear sides.

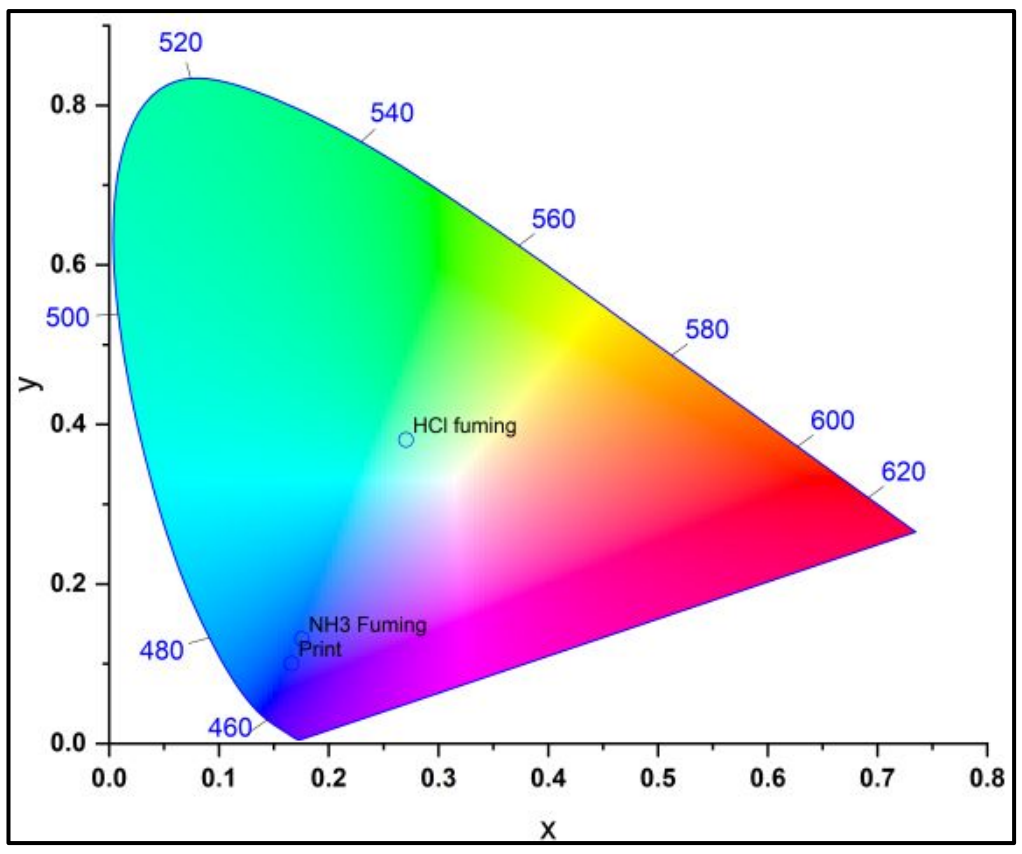

SF 19: Chromaticity plot of print, and HCl fumed & NH_3 fumed prints; λ_{ex} = 390 nm.

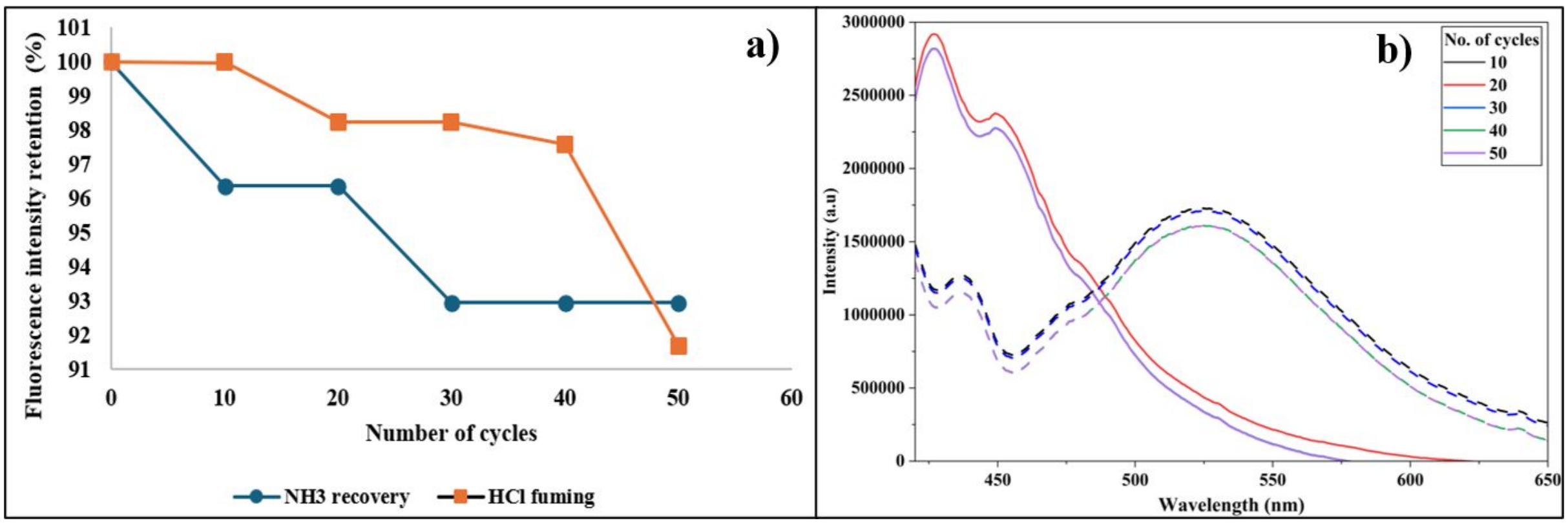

SF 20: a) Fluorescence intensity retention of the HCl-fumed and NH_3-recovered printed patterns during 50 consecutive HCl/NH_3 vapor switching cycles and b) PL spectra of the HCl-fumed (solid lines) and NH_3-recovered (dashed lines) printed patterns recorded after 10, 20, 30, 40, and 50 switching cycles.

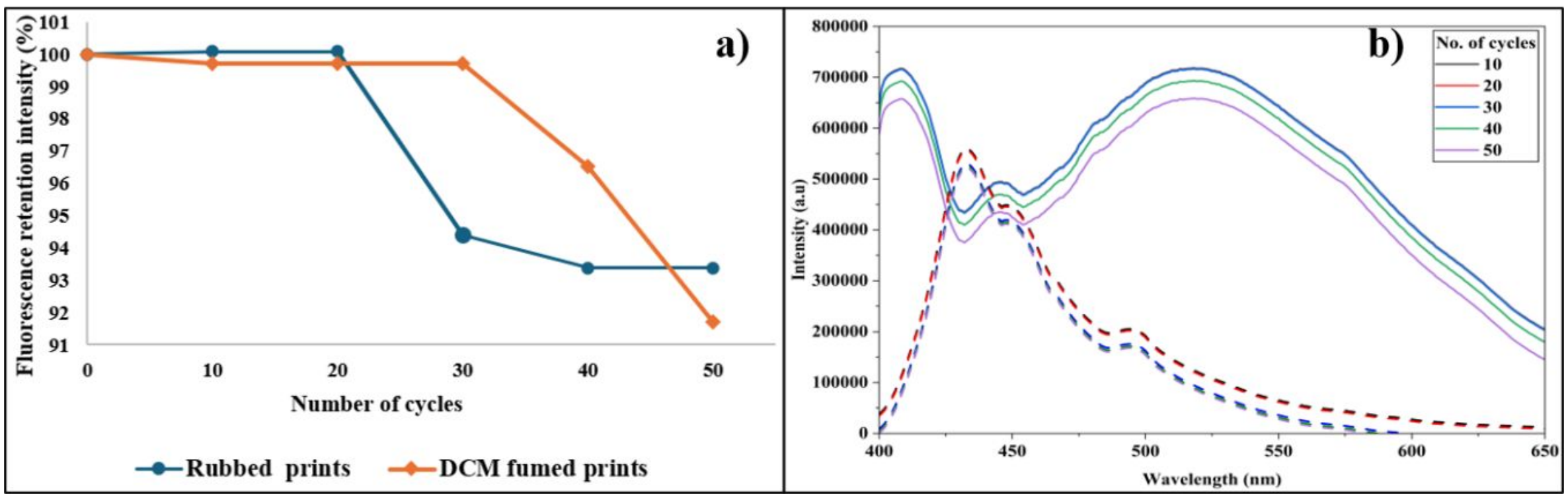

SF 21: a) Fluorescence intensity retention of the rubbed and DCM-vapor-recovered prints during 50 consecutive mechanofluorochromic switching cycles and b) PL spectra of the rubbed (solid lines) and DCM-vapor-recovered (dashed lines) printed patterns recorded after 10, 20, 30, 40, and 50 switching cycles.

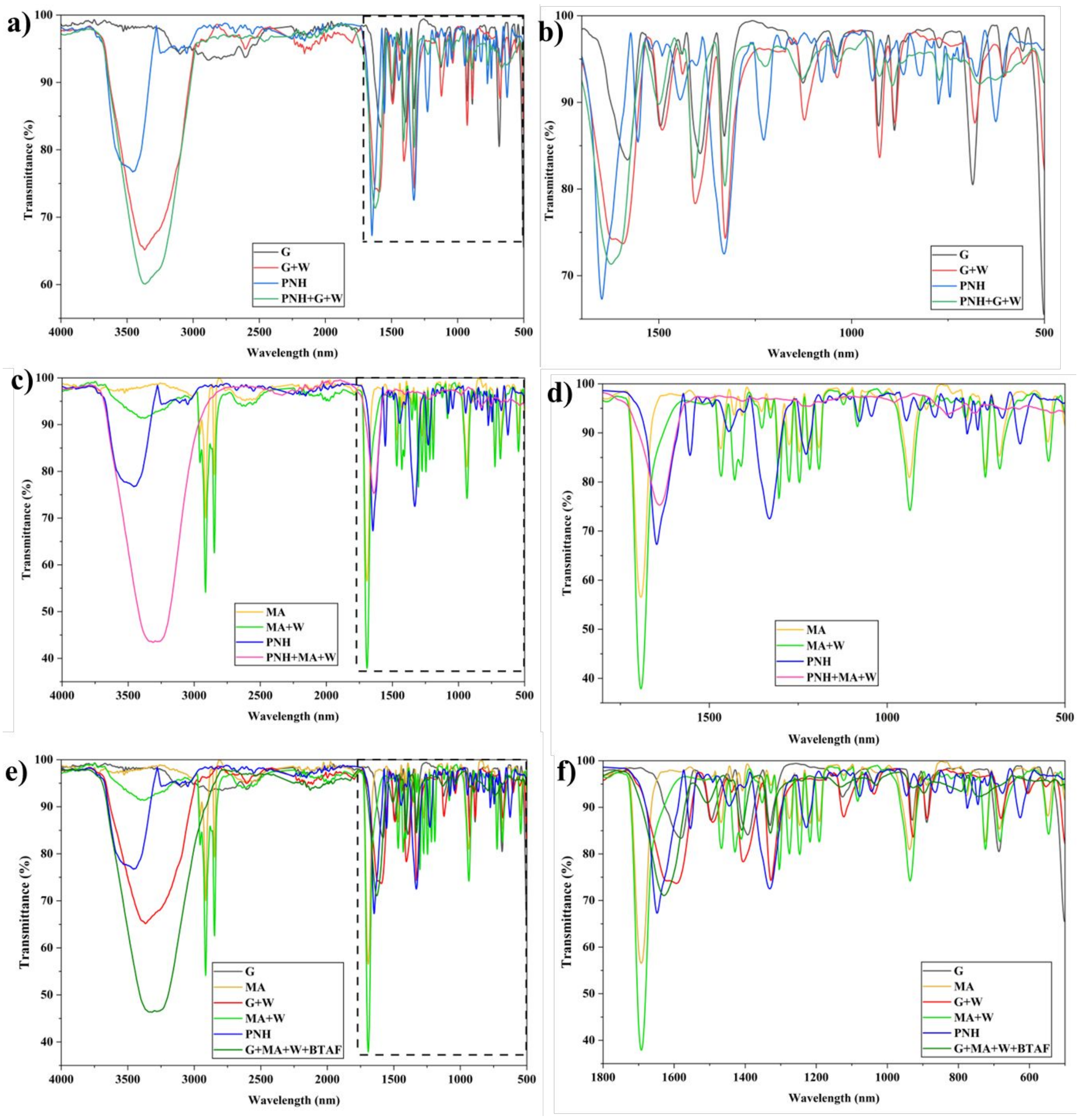

SF 22: FTIR spectral comparison of a) glycine, water, glycine + water, PNH, and glycine + water + PNH, b) enlarged view of the highlighted spectral region in (a), c) myristic acid, water, myristic acid + water, PNH, and myristic acid + water + PNH, (d) enlarged view of the highlighted spectral region in (c), e) glycine, water, glycine + water, myristic acid + water, PNH, and glycine + myristic acid + water + PNH; and (f) enlarged view of the highlighted spectral region in (e). G- Glycine, MA- Myristic acid, W- Water.

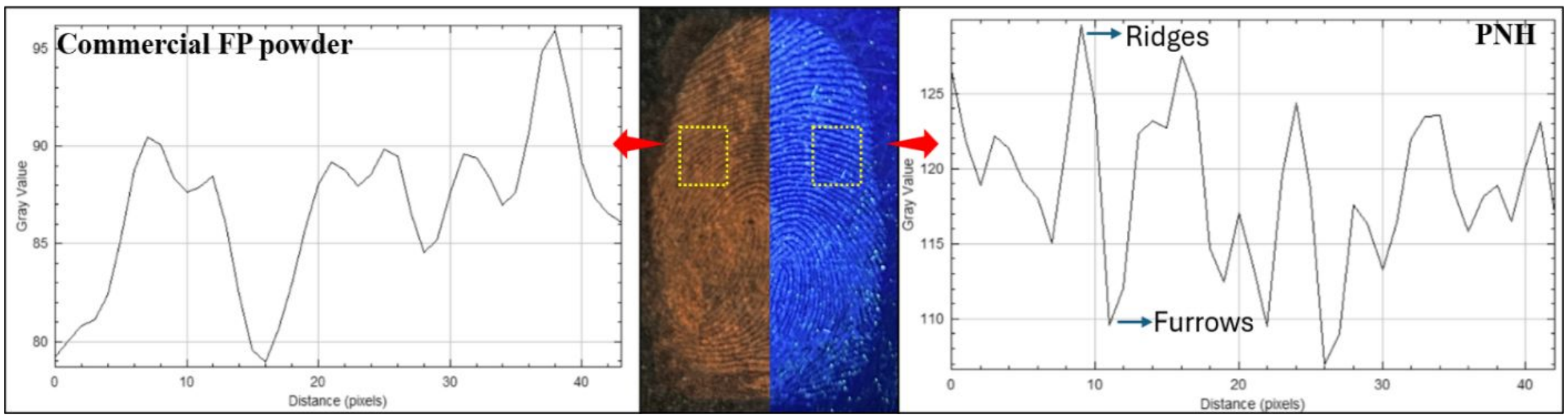

SF 23: Comparison of LFP visualization using a commercial fingerprint powder and PNH under UV illumination, along with the corresponding grayscale intensity profiles.

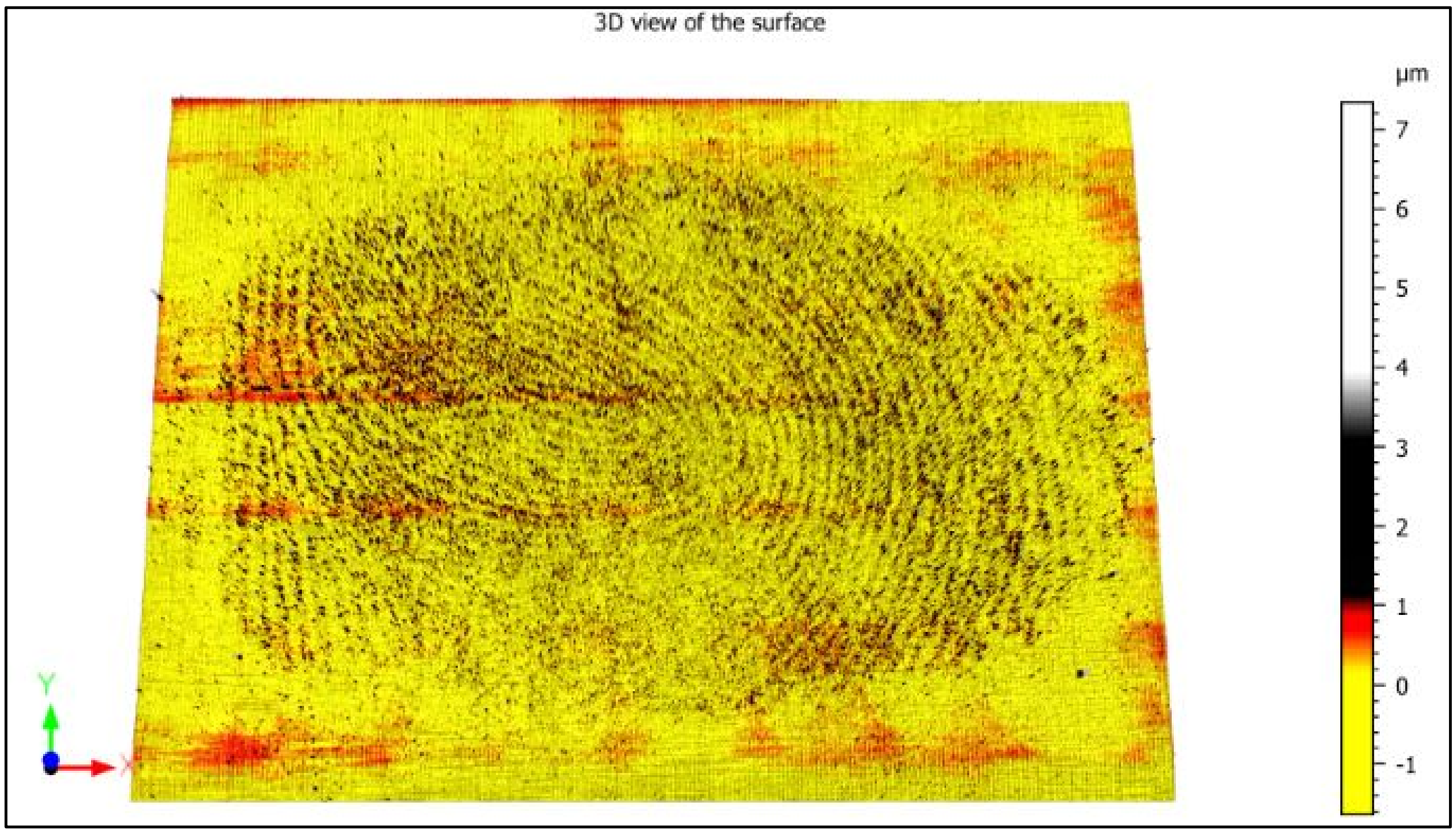

SF 24: 3D surface profilometry image of a LFP developed on a CD surface.

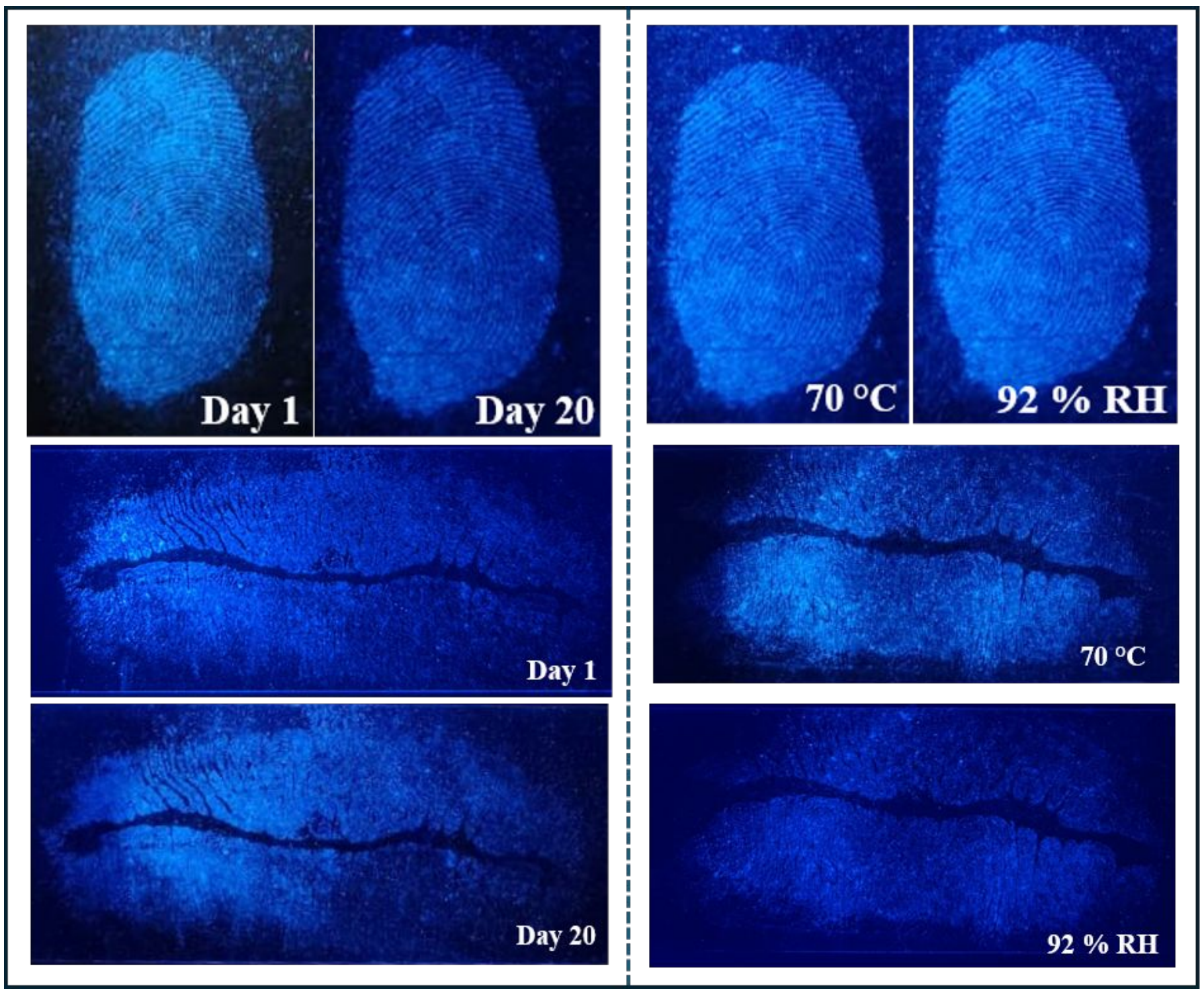

SF 25: Development of aged LFPs and LLPs using PNH under different storage and environmental conditions.

ST 3: Comparison of PNH with recently reported multifunctional fluorescent materials for anti-counterfeiting and forensic applications.

**Material**	**Solid-state λ_{em} (nm), Quantum yield**	**Mechano-fluorochromism (shift/recovery)**	**Pressure sensitivity**	**Acido-fluorochromism (reversibility)**	**Cycling stability**	**Photostability**	**LFP levels visualized**	**Ref**
PNH	425 nm and 449 nm, Not reported	430/450 → 440/560 nm (blue→orange); recovered with DCM vapor	0.018 - 0.088 MPa	425/449 → 453/532 nm with HCl; restored with NH_3	50 HCl/NH_3 cycles; 50 mechano/DCM cycles	12 h continuous UV (10 W) - intensity unchanged	Levels 1-3 on non-porous, semi-porous, CD, same-background white tile	This work
FNH (fluorene-naphthalene Schiff base)	Cyanish-green under 420-480 nm, Not reported	Rub-induced; reversible with DCM	Not reported	NaOH/KOH-sensitive (ink film)	Not reported	Good photostability of prints	Levels 1-2 detailed	[1]

BTNH (benzothiophene-hydroxy naphthalene)	Excitation-dependent blue→greenish-yellow emission 260-480 nm, Not reported	Rub-induced; DCM-reversible	Not reported	pH-sensitive (reversible in print)	Not reported	Good lightfastness of prints	Level 1-3 on non-porous, Levels 1-2 on semi-porous, under daylight	[2]
BTAF (fluorene–bithiophene)	510 nm, Quantum yield= 31.57%	Not reported	Not reported	Not explicitly stated	Not reported	Continuous printing durability	Levels 1-3 on diverse substrates	[3]
CNS (carbazole-naphthalene Schiff base)	450 nm, Not reported	Rub-induced orange fluorescence from weak blue fluorescence	Not reported	NaOH-triggered color change in print	Not reported	Good adherence/rub resistance	Levels 1-3 on non-porous and semi-porous	[4]
OAF (fluorene-vanillin Schiff base)	510 nm, Quantum yield= 7.9%	Not reported	Not reported	Not reported	Not reported	Photobleaching/scuffing resistant offset prints	Levels 1-3 on non-porous, semi-porous, leather, leaf, multicolored CD	[5]
TPA-NC	530 nm, 19.02 %	530 → 560 nm (30 nm red shift); reversible on recrystallization	Not reported	Not reported	Not reported	Water-based flexo-ink prints	Not reported	[6]
Zn Schiff base HL	457 nm, Not reported	457 → 545 nm (blue→green); acetonitrile-reversible	Not reported	Only complexes 1-THF and 1-DMF	Not reported	Not quantified	Not reported	[7]
TBBH and TBIH	375-418 nm and 374-406 nm in different	Reversible mechanofluochromism with AIEgen	Not reported	Protonation-driven switching	Not reported	Not quantified	Not reported	[8]

	conc. of THF, Quantum yield= 23.4% and 21.6%							
PySS and PySP	509 nm and 520 nm; Quantum yield= 24.13 % and 19.01%	Reversible under force/solvent	12.5 ton hydraulic press	Not reported	Not reported	Rewritable papers demonstrated	Not reported	[9]

References

[1] Muthamma, K.; Acharya, S.; Sunil, D.; Shetty, P.; et al. Fluorene-Naphthalene Schiff Base as a Smart Pigment in Invisible Ink with Multiple Security Features for Advanced Anticounterfeiting and Forensic Applications. J. Colloid Interface Sci. 2024, 653, 209–219. https://doi.org/10.1016/j.jcis.2023.09.033.

[2] S. N., A.; Jain, R. K.; P. J., A.; Sunil, D.; Kashyap, N. N. Benzothiophene-Hydroxy Naphthalene Hybrid: An Investigation of Fluorescence Features for Advanced Security and Forensic Applications. Surf. Interfaces 2025, 68, 106738. https://doi.org/10.1016/j.surfin.2025.106738.

[3] Jain, R. K.; et al. Multifunctional Fluorophore Based on Fluorene–Bithiophene Architecture for Latent Print Visualization and Stimuli-Responsive Eco-Friendly Security Inks. Prog. Org. Coat. 2026, 213, 109995. https://doi.org/10.1016/j.porgcoat.2026.109995.

[4] Mehta, R.; Jain, R. K.; Sunil, D.; P. J., A. Carbazole-Naphthalene Based Colorant with Rub-Induced and Alkali-Sensitive Fluorescence for Security Printing and Latent Fingerprint Development. J. Mol. Struct. 2025, 1337, 142279. https://doi.org/10.1016/j.molstruc.2025.142279.

[5] Jain, R. K.; et al. Fluorene-Vanillin Hybrid: A Multifunctional Fluorophore for Latent Fingerprint Detection and Sustainable Offset Security Ink Development. Prog. Org. Coat. 2025, 207, 109425. https://doi.org/10.1016/j.porgcoat.2025.109425.

[6] Muthamma, K.; et al. Mechano- and Solvato-Fluorochromic Chalcone and Its Water-Based Flexo-Ink for Anticounterfeit Applications. Prog. Org. Coat. 2023, 184, 107832. https://doi.org/10.1016/j.porgcoat.2023.107832.

[7] Liang, Q.-F.; Zheng, H.-W.; Yang, D.-D.; Zheng, X.-J. Zn(II) Complexes Based on a Schiff Base: Mechanochromism- and Solvent Molecule-Dependent Acidochromism. Cryst. Growth Des. 2022, 22, 3903–3913. https://doi.org/10.1021/acs.cgd.2c00320.

[8] Jia, J.; Wu, L. Stimuli-Responsive Fluorescence Switching: Aggregation-Induced Emission (AIE), Protonation Effect, and Reversible Mechanofluorochromism of Tetraphenylethene Hydrazone-Based Dyes. Org. Electron. 2020, 76, 105466. https://doi.org/10.1016/j.orgel.2019.105466.

[9] Bhatta, R. P.; Sumit; Kachwal, V.; Vishwakarma, V.; Roy Choudhury, A.; Laskar, I. R. Precise Molecular Design for a Twisted Pyrene-Thiophene Based Mechanofluorochromic Probe with Large Stokes Shift and Feasibility Study towards Security Ink and Re-writable Papers. J. Mater. Chem. C 2024, 12, 12906–12918. DOI: 10.1039/D4TC02262A.
